# Entanglement of spin transition and structural adaptability: manipulating the slow spin equilibrium by the guest-mediated fine-tuning of elastic frustration

**DOI:** 10.1039/d5sc01202c

**Published:** 2025-07-21

**Authors:** Yuqiao Chai, Yu-Ting Yang, Jin-Peng Xue

**Affiliations:** a School of Material and Chemical Engineering, Ningbo University of Technology Ningbo Zhejiang 315211 China; b School of Materials Science and Chemical Engineering, Ningbo University Ningbo 315211 China xuejinpeng@nbu.edu.cn; c Jiangxi Provincial Key Laboratory of Functional Crystalline Materials Chemistry, Jiangxi University of Science and Technology Ganzhou 341000 Jiangxi China

## Abstract

A comprehensive analysis of physical and chemical properties using the same family of spin-crossover complexes is crucial for understanding and designing structure–property relationships. However, finding an appropriate system remains challenging. Here, a series of guest-saturated states based on the 2D Hofmann-type framework [Fe^II^(prentrz)_2_Pd^II^(CN)_4_]·guest (prentrz = (1*E*,2*E*)-3-phenyl-*N*-(4*H*-1,2,4-triazol-4-yl)prop-2-en-1-imine; 1·guest) is reported, exhibiting a guest-manipulated slow dynamic effect on spin equilibrium in an incomplete two-step spin-crossover (SCO) process. Using a full-sealed method by modulating the mixing ratios and types of CH_3_OH, H_2_O, and D_2_O, the stable maintenance of guest-saturated states allows fine-tuning of the elastic frustration (*ξ*) of the framework to realize SCO behaviors in the unexplored region between one-step incomplete (HS_0.5_LS_0.5_ ↔ HS) and two-step complete (LS ↔ HS_0.5_LS_0.5_ ↔ HS) processes. In the semi-sealed method, guest molecules gradually escape from the material until the guest-saturated state disappears. This continuous loss shifts the slow spin equilibrium from a state that is difficult to switch to one that fully completes the transition. The study demonstrates that guest molecule modulation is more controllable than structural deformation effects on elastic frustration, offering a pathway to discover hidden types of SCO materials and develop new stimulus-responsive materials.

## Abstract 2

A guest-controlled strategy enables the precise tuning of slow spin equilibrium in Hofmann-type SCO frameworks, allowing bidirectional modulation between incomplete and two-step complete SCO by varying the ratios and types of guest solvents.

## Graphical abstract

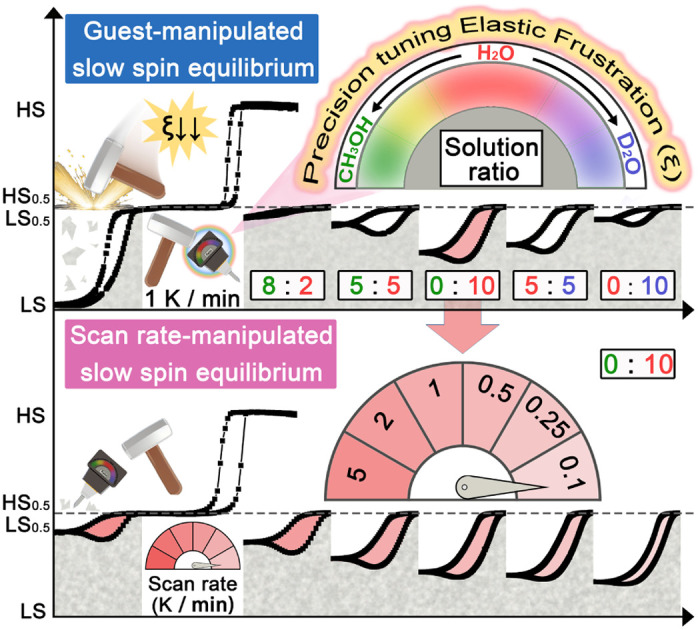

## Introduction

Multistable materials that change structure and functionality in response to external stimuli have attracted increasing attention for their suitability for analyzing phase transition mechanisms and their potential applicability to molecular sensors and switches.^1–5^ Among them, spin-crossover (SCO) materials have garnered extensive study due to their potential for multi-input multi-output characteristics. These materials respond to various stimuli, such as light, heat, pressure, and guest molecules, and exhibit diverse output properties, including changes in magnetism, color, dielectric constants, and mechanical properties.^6–10^ As with all multistable materials, the ultimate goal in the design of SCO materials is to achieve manipulability, entailing optimal or programmable performance during the reversible transition between high-spin (HS) and low-spin (LS) states.^11^ The occurrence of SCO behavior requires suitable ligand field strengths, as exemplified by a series of mononuclear {Fe^II^N_6_} SCO compounds that have been rationally structured to enable a comprehensive analysis of hysteresis properties, with theoretical calculations confirming that the growth of the hysteresis loop is controlled by electrostatic contributions.^12^ In addition, the integral nature of spin-crossover (SCO) behavior is determined by the elastic cooperativity between metal centers, which is closely related to host–host and host–guest interactions.^13–16^ For example, polymorphism in SCO compounds typically exhibits different SCO properties with varying transition temperatures and numbers of steps.^17,18^ Additionally, the reversible modulation of SCO properties by different guest molecules is achieved through mechanisms such as lattice strain, hydrogen-bonding interactions, electrostatic interactions, and ligand substitution.^19,20^ In addition to the study of conventional properties like multi-step and abrupt SCO with wide hysteresis, unusual spin transitions that significantly depend on the temperature-scan rate under strong cooperativity—known as slow spin equilibrium or the kinetic trapping effect—have also garnered significant attention.^21–24^ SCO complexes exhibiting slow spin equilibrium possess desirable properties, including the highest reported values for temperature-induced excited spin-state trapping (TIESST, *T*_TIESST_ = 250 K) and long-lived light-induced excited spin-state trapping (LIESST, *T*_LIESST_ = 80 K).^25–27^ Although slow spin equilibrium often occurs in SCO compounds with long alkyl chains, modification with long-chain alkanes does not inevitably result in the kinetic trapping effect in SCO materials.^28–39^ Consequently, compared to the comprehensive analysis of conventional SCO behavior in a series of well-characterized compounds, the study of slow spin equilibrium presents challenges in identifying an appropriate SCO system with similar configurational and stacked structures.

Metal–organic frameworks (MOFs), known for their high tunability, offer a versatile platform for exploring various properties, especially those related to cooperativity.^40–42^ The intrinsic porosity of SCO-MOFs allows for the modulation of host–guest interactions through guest molecule substitutions, thereby fine-tuning the coordination environment of metal centers.^43,44^ In certain SCO-MOFs, the SCO behavior is highly sensitive to the guest molecule, leading to significant variations in the numbers of steps, hysteresis, and transition temperatures (*T*_1/2_) without substantially altering the structure of the framework.^45–48^ Recent computational studies utilizing Monte Carlo (MC) simulations have revealed that the degree of elastic frustration (*ξ*) has been employed to investigate multi-step spin transitions in a rigid 2D lattice.^49^ A “frozen” anomaly (*ξ* = 1.045) was identified at the junction between incomplete (HS_0.5_LS_0.5_ ↔ HS) and complete two-step (LS ↔ HS_0.5_LS_0.5_ ↔ HS) spin transitions, which is hypothesized to result from kinetic effects. This “frozen” anomaly implies that a framework with a specific *ξ* value possesses the potential to undergo SCO with slow spin equilibrium. Notably, this suite of theoretical models and the concept of elastic frustration have been effectively applied in analyzing anti-ferroelastic and ferroelastic interactions within Hofmann-type SCO-MOFs.^50–57^ The results suggest that there exists a suitable Hofmann-type framework with a *ξ* value near the “frozen” anomaly, potentially imparting a slow dynamic effect on SCO behavior.

Previous studies, including our own, have demonstrated that the 2D Hofmann-type MOF [Fe^II^(prentrz)_2_Pd^II^(CN)_4_] (prentrz = (1*E*,2*E*)-3-phenyl-*N*-(4*H*-1,2,4-triazol-4-yl)prop-2-en-1-imine, 1) exhibited pore-adjustable behavior, with the *ξ* values of the framework fluctuating around 1 according to the SCO behavior.^58,59^ Notably, CH_3_OH guest molecules in as-grown crystals tend to vacate the framework when exposed to atmospheric conditions, rapidly being lost and replaced by H_2_O molecules. The distinct interactions of compound 1·guest with H_2_O and CH_3_OH molecules have established that CH_3_OH molecules within the pores increase the *ξ* value of the framework.^48,60^ Nonetheless, 1·guest compounds can remain stable in dual-guest form, containing both CH_3_OH and H_2_O molecules, when immersed in H_2_O–CH_3_OH mixed solvents. In this state of maximum guest uptake, the compounds are considered to be in a guest-saturated state. In this work, guest-saturated states of 1·guest are maintained through immersion in proportional solvents of CH_3_OH, H_2_O, and D_2_O, exhibiting a guest-manipulated slow dynamic effect of spin equilibrium in an incomplete two-step SCO process. The immersion employs full-seal and semi-sealed methods. By using the full-seal method to modulate the mixing ratios and types of CH_3_OH, H_2_O, and D_2_O, three types of mixed solvents are formed: H_2_O–CH_3_OH, D_2_O–CH_3_OH, and H_2_O–D_2_O. The stable maintenance of guest-saturated states allows for fine-tuning of the elastic frustration of the framework, thereby first enabling a varying degree of slow spin equilibrium using the same temperature-scan rate and framework. The semi-sealed method is employed to gradually desorb the guest-saturated state using three H_2_O–CH_3_OH ratios (2 : 8, 8 : 2, 10 : 0) through *in situ* heating. The continuous loss of guest molecules reveals a transition from a slow, hindered spin equilibrium to fully responsive state, ultimately resulting in the disappearance of the slow equilibrium behaviour. Manipulating the slow spin equilibrium by two types of guest-mediated methods provides comprehensive insights into the role of guest molecules in fine-tuning elastic frustration and SCO behaviors, offering underlying mechanisms in the unexplored region between one-step incomplete (HS_0.5_LS_0.5_ ↔ HS) and two-step complete (LS ↔ HS_0.5_LS_0.5_ ↔ HS) SCO behaviors in such Hofmann-type SCO-MOFs.

## Results and discussion

### Crystal structures of 1·3H_2_O·3/2CH_3_OH and the deduced sequence of guest distribution modulation

**Fig. 1:**
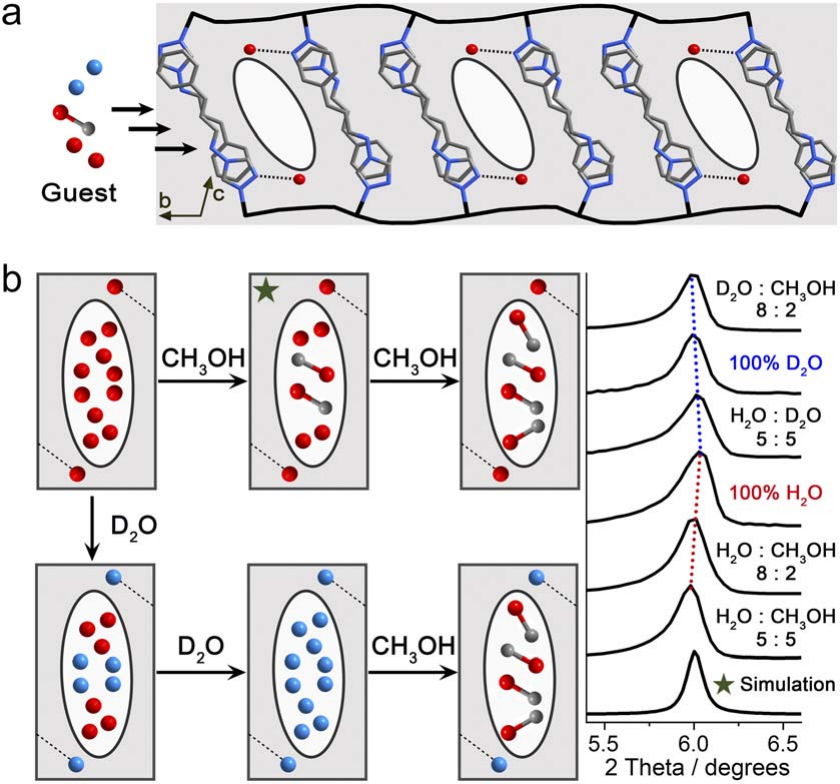
(a) The pore configuration and interlayer interactions of the guest-saturated state 1·guest viewed along the *a*-axis direction. The black lines represent the Fe[Pd(CN)_4_] 2D network constructed from [Fe–N≡C–Pd]_*n*_. All hydrogen atoms were omitted for clarity. (b) The distribution of H_2_O, D_2_O, and CH_3_OH in different guest-saturated states inferred from the PXRD patterns after guest exchange, and SC-XRD data for 1·3H_2_O·3/2CH_3_OH. The green star indicates the reduced structure and PXRD simulation of 1·3H_2_O·3/2CH_3_OH.

Due to the tendency for crystal fragmentation during spin transitions, a relatively small crystal of the guest-saturated state 1·3H_2_O·3/2CH_3_OH was selected from samples immersed in a solution with a 5 : 5 H_2_O–CH_3_OH ratio. This selected single crystal was further sealed in a quartz tube with the same mixed solution for single-crystal X-ray diffraction (SC-XRD) measurements. The compound 1·3H_2_O·3/2CH_3_OH crystallizes in the triclinic space group *P*1̄ at temperatures of 293, 190, 150, 130, 120, and 85 K, respectively (Table S1). The asymmetric unit of 1·3H_2_O·3/2CH_3_OH remains consistent at all temperatures, containing two crystallographically independent Fe ions (Fe_1_ and Fe_2_) situated at inversion centers, two prentrz ligands with distinct conformations (conformer 1 for Fe_1_ and conformer 2 for Fe_2_), three H_2_O molecules, two-thirds of a CH_3_OH guest molecule, and one [Pd(CN)_4_]^2−^ bridging ligand (Fig. S1 and S2). The guest-saturated state 1·3H_2_O·3/2CH_3_OH corresponds to the large channel-type pore (lcp) phase described in previous work, exhibiting the same 2D layer, supramolecular structure, and channel pores extending along the crystallographic *a*-axis direction (Fig. 1a, S3 and S4).^57^

Based on the distribution of CH_3_OH and H_2_O molecules in the pores of compound 1·3H_2_O·3/2CH_3_OH, it can be inferred that CH_3_OH molecules preferentially replace the H_2_O molecules and occupy the center of the channel-type pore (Fig. 1b and S5). When immersed in pure CH_3_OH solution for a week, the samples turned off-white and lost crystallinity. This supports the hypothesis that the weak O–H⋯N hydrogen bond between the prentrz ligand (N_3_) and a H_2_O (O_5_) molecule plays a crucial role in stabilizing the framework structure (Fig. S6). PXRD analyses were performed to examine the expansion/contraction of the framework in different guest-saturated states (Fig. 1c and S7). H_2_O and D_2_O, with precisely the same kinetic diameters (2.64 Å), as well as CH_3_OH, were used to gradually substitute the original guest molecules in the pore cavity to achieve a gradient effect. The principal diffraction peak in the low-angle region of the guest-saturated samples, identified as (001), undergoes a peak position shift attributed to the displacement of guest molecules. This indicates that the 2D network in the [110] direction undergoes a certain degree of expansion/contraction. Starting with the guest-saturated sample with a 10 : 0 H_2_O–CH_3_OH ratio (pure H_2_O), an increase in the proportion of CH_3_OH causes the characteristic peak to shift to a lower angle, indicating framework expansion. This indirectly suggests the disruption of the hydrogen-bonding network among H_2_O molecules. Replacing H_2_O with D_2_O results in the characteristic peak, as well as all major peaks, shifting to lower angles, indicating the expansion of the framework in all dimensions. This finding contrasts with the common understanding that deuterium substitution typically leads to stronger hydrogen bonds and a more contracted structure. Such structural expansion may arise from the rearrangement of donor–acceptor distances in the O–H⋯N (H_2_O and host structure) and O–H⋯O (H_2_O and H_2_O) hydrogen bonds within the pore cavity, *i.e.*, isotopic polymorphism and the geometric H/D isotope effect (GIE).^61,62^ Further replacing D_2_O with CH_3_OH continues to shift the characteristic peak to lower angles, suggesting the effect on increasing the *ξ* value of the framework follows the order: CH_3_OH > D_2_O > H_2_O (Fig. S8).

**Fig. 2:**
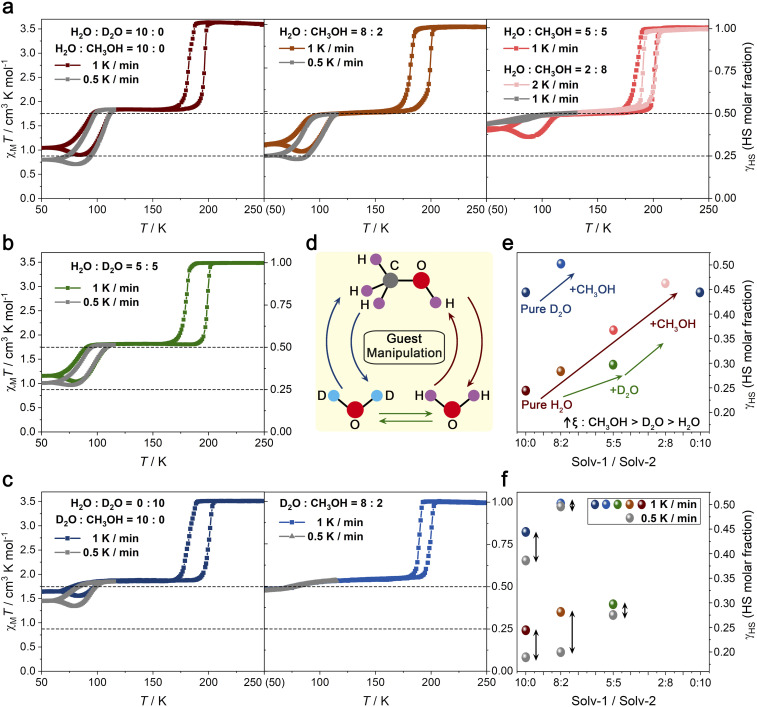
Temperature-dependent *χ*_M_*T* and *γ*_HS_ values of guest-saturated samples with varying solvent types and ratios. (a) The two-step SCO for guest-saturated samples with H_2_O–CH_3_OH ratios of 2 : 8 (1 and 2 K min^−1^), 5 : 5 (1 K min^−1^), 8 : 2 (1 and 0.5 K min^−1^), and 10 : 0 (1 and 0.5 K min^−1^). (b) The two-step SCO of a guest-saturated sample with a 5 : 5 (1 and 0.5 K min^−1^) H_2_O–D_2_O ratio. (c) The two-step SCO of guest-saturated samples with D_2_O–CH_3_OH ratios of 10 : 0 (1 and 0.5 K min^−1^) and 8 : 2 (1 and 0.5 K min^−1^). (d) A schematic diagram of the cyclic substitution of H_2_O, D_2_O, and CH_3_OH guest molecules. (e) A plot of min. *γ*_HS_ value *versus* Solv-1/Solv-2 ratio at a scan rate of 1 K min^−1^. (f) A plot of min. *γ*_HS_ value *versus* Solv-1/Solv-2 ratio at 0.5 K min^−1^ compared to 1 K min^−1^. The *x*-axes of (e) and (f) represent the solvent ratios described in (a–c); dark to light red points denote H_2_O–CH_3_OH ratios of 10 : 0, 8 : 2, 5 : 5, and 2 : 8, respectively; the green point shows a 5 : 5 H_2_O–D_2_O ratio; the dark and light blue points represent D_2_O–CH_3_OH ratios of 10 : 0 and 8 : 2, respectively; and the gray points give the corresponding ratios at 0.5 K min^−1^.

The variable-temperature magnetic susceptibilities of guest-saturated samples with 2 : 8, 5 : 5, 8 : 2, and 10 : 0 H_2_O–CH_3_OH ratios were first measured using the full-sealed method, directly probing the guest-exchange influence on the slow spin equilibrium of Fe_2_ (Fig. 2a and S9–S11). Each sample shows χ_M_*T* values of 3.48–3.63 cm^3^ K mol^−1^ above 210 K, consistent with a complete HS phase (*γ*_HS_ = 1). Upon cooling at 1 K min^−1^, the *γ*_HS_ values drop rapidly to around 0.5 at 190 K, after which a gradual decline continues until 113 K. Further cooling reveals a guest-manipulated slow spin equilibrium for Fe_2_—with *γ*_HS_ values decreasing from 0.44 (2 : 8) to 0.41 (5 : 5), 0.32 (8 : 2), and 0.28 (10 : 0) at 50 K, indicating that reducing the methanol content enhances the effectiveness in overcoming the slow spin equilibrium of Fe_2_, as alcohols generally impede the flexibility (↑*ξ*).^59^ The *γ*_HS_ values of 0.25 (8 : 2) and 0.22 (10 : 0) at 50 K at a scan rate of 0.5 K min^−1^ exhibit higher SCO completeness compared to those at 1 K min^−1^, indicating that the scan-rate-manipulated slow spin equilibrium persists in the guest-saturated samples with a dominant water proportion. During warming, at 1 K min^−1^, the *γ*_HS_ values unexpectedly drop at specific temperatures (65 K for 5 : 5, 60 K for 8 : 2, and 61 K for 10 : 0), reaching minimum values of 0.36 (5 : 5) at 88 K, 0.28 (8 : 2) at 84 K, and 0.26 (10 : 0) at 84 K. Upon heating, the *γ*_HS_ values recover (Fig. S11). Meanwhile, the reversibility of the spin equilibrium in the second-step spin transition was evaluated using a guest-saturated sample (10 : 0 H_2_O–CH_3_OH ratio) at 1 K min^−1^. Three successive thermal cycling procedures were performed between 50 and 120 K, revealing that the temperature-dependent magnetic susceptibilities of the sample showed stable reversibility (Fig. S12). The SCO of Fe_1_ in the guest-saturated state 1·3H_2_O·3/2CH_3_OH (5 : 5 H_2_O–CH_3_OH ratio) occurs at the first-step spin transition, accompanied by a change in the average Fe1–N bond length from 2.156(5) Å at 293 K to 1.959(5) Å at 190 K (Δ*d*_Fe–N_ = 0.197 Å)^63^ and a contraction of the unit-cell volume by 4.4% (Fig. S13, Tables S2 and S3). The average Fe_2_–N average bond length remains essentially constant over the temperature interval of 70 K: 2.162(5) Å at 190 K, 2.161(5) Å at 150 K, 2.162(5) Å at 130 K, and 2.166(6) Å at 120 K. A reduction in temperature to 85 K witnesses a slight decrease in the average Fe_2_–N bond length to 2.138(13) Å, representing the occurrence of possible SCO for Fe_2_ (Δ*γ*_HS_ = 12.2%), while further completeness is limited by the temperature control system.

Replacing CH_3_OH with CH_3_CH_2_OH as the guest molecule caused the guest-saturated sample (5 : 5 H_2_O–CH_3_CH_2_OH ratio) to exhibit incomplete one-step SCO, without evidence for a 2nd-step spin transition or slow spin equilibration (Fig. S14). Similarly, introducing *N*,*N*-dimethylformamide (DMF) as the guest molecule suppressed the SCO behavior in guest-saturated samples with 1 : 9 and 5 : 5 H_2_O–DMF ratios, and these remain in the HS state over the 50–250 K temperature range (Fig. S15). At a lower DMF content (9 : 1 H_2_O–DMF), gradual and incomplete one-step SCO was observed (Fig. S16). These results demonstrate that the slow spin equilibration observed in the second-step transition is highly sensitive to the structural and electronic characteristics of the guest molecules. To further investigate whether this sensitivity arises from factors beyond molecular size and general physical properties, two types of mixed solvents containing D_2_O were used to manipulate the SCO behaviour of guest-saturated samples: H_2_O–D_2_O mixtures with ratios of 10 : 0 (Fig. 2a), 5 : 5 (Fig. 2b), and 0 : 10 (Fig. 2c); and D_2_O–CH_3_OH mixtures with ratios of 10 : 0, and 8 : 2 (Fig. 2c and S17–S21). The *γ*_HS_ values of the guest-saturated samples with different H_2_O–D_2_O ratios reached 0.28 (10 : 0), 0.33 (5 : 5), and 0.470 (0 : 10) at 50 K during the cooling process at 1 K min^−1^ (Fig. S17 and S18). In the warming process, the *γ*_HS_ values of the three ratio samples started to decrease anomalously at 61 K, reaching 0.26 (10 : 0) at 84 K, 0.29 (5 : 5) at 81 K, and 0.467 (0 : 10) at 81 K. The slow spin equilibrium of Fe_2_ in structures with pure D_2_O is more difficult to thermally overcome than those with pure H_2_O at scan rates of either 1 or 0.5 K min^−1^ (Fig. S19a). For guest-saturated samples with varying D_2_O–CH_3_OH ratios, replacing 20% of the pure D_2_O solution with CH_3_OH resulted in the spin transition of Fe2 being unaffected by changes in scan rate (Fig. S19b). The SCO behavior of the sample with an 8 : 2 D_2_O–CH_3_OH ratio is similar to that observed in the 2 : 8 H_2_O–CH_3_OH sample, which contains a higher proportion of CH_3_OH, and contrasts with the behavior seen in the 8 : 2 H_2_O–CH_3_OH sample (Fig. 2d, e and S20). The more difficult it is to thermally overcome the slow spin equilibrium, the less it is affected by changes in scan rate, and the greater the *ξ* value corresponding to the framework, which is consistent with the abnormal expansion of the framework with D_2_O observed *via* PXRD (Fig. 2f).

To further verify the relationship between guest-modulated spin equilibrium dynamics and the structural and electronic properties of guest molecules, time-resolved *in situ* micro-Raman spectroscopy experiments (spot diameter ∼1 mm) were performed on guest-saturated samples prepared with pure H_2_O and D_2_O (Fig. S22–S24). As shown in Fig. S23, structural responses were tracked by monitoring the characteristic bands at around 1580 cm^−1^ (C=N stretch modes) and 1620 cm^−1^ (C=C stretch modes) of the prentrz ligands. Upon the incremental addition of CH_3_OH to H_2_O- or D_2_O-saturated samples, these characteristic bands initially remained unchanged, but subsequently exhibited a discernible redshift. This redshift signifies the progressive displacement of H_2_O/D_2_O in the pores by CH_3_OH, leading to enhanced structural flexibility and the weakening of the hydrogen-bonding network, ultimately resulting in the expansion of the pore microstructure. In contrast, when D_2_O-saturated samples were exposed to increasing and excessive amounts of H_2_O, the positions of the two bands remained unchanged, indicating that isotopic substitution alone exerts only a limited effect on the hydrogen-bonding network (Fig. S24). Therefore, the range of modulation of spin equilibrium in samples with varying H_2_O–D_2_O ratios remains restricted compared with that achieved after CH_3_OH incorporation. Meanwhile, the O–H⋯N hydrogen bond distance in 1·3H_2_O·3/2CH_3_OH is 2.904(4) Å at 293 K, indicating that the samples with guest-saturated states are weak H-bonded compounds (D: proton donor, A: proton acceptor, *d*_D⋯A_ > 2.90 Å). However, minute shifts in the H-bonding distance can be propagated cooperatively through the crystal lattice, with deuteration effects influencing the physical and chemical properties of the materials (Table S3).^64^ The thermal hysteresis loops of Fe_1_ fluctuate with changes in the immersion solvent system, directly indicating the involvement of guest molecules in the strong cooperative interactions between the metal centers.^56,59^ The SCO behavior of the framework, particularly the slow spin equilibrium of Fe_2_, exhibits high sensitivity and can be manipulated by the guest molecules, including CH_3_OH, H_2_O, and D_2_O. Additionally, hydrogen-bonding distances decrease upon cooling (Table S3), suggesting ferroelastic interactions between the host and guest.^45^

**Fig. 3:**
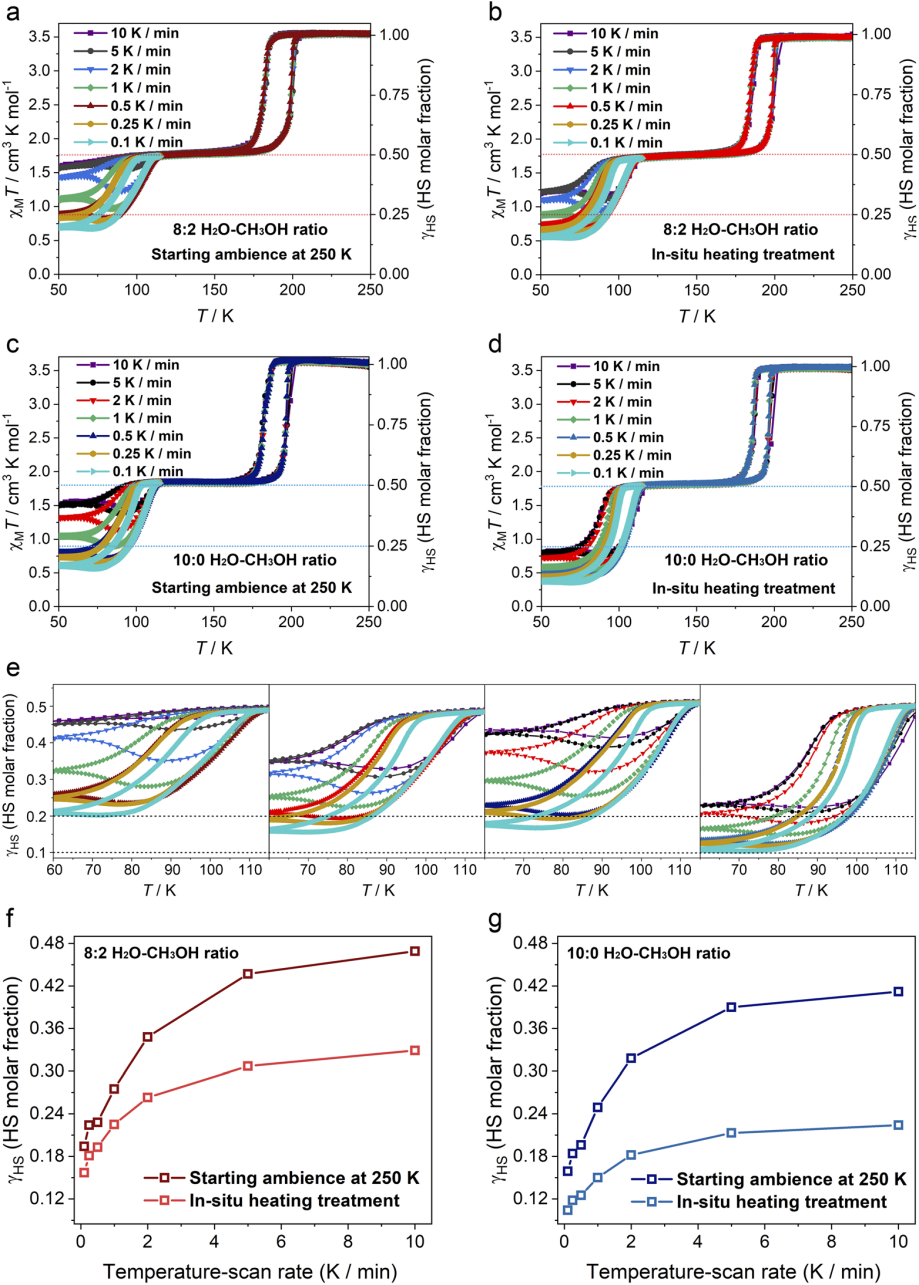
A study of χ_M_*T* and *γ*_HS_*versus T* for guest-saturated samples with 8 : 2 and 10 : 0 H_2_O–CH_3_OH ratios at varying scan rates (0.1–10 K min^−1^) and with varying guest effects (intact and partially guest-depleted states). (a) Magnetic susceptibilities of a fresh sample with an 8 : 2 H_2_O–CH_3_OH ratio were measured, with a starting ambient temperature of 250 K, at various scan rates. (b) After *in situ* warming to 300 K and holding for 10 min, the magnetic susceptibilities of the partially guest-depleted state were measured after cooling to 250 K for cooling–warming cycles. Magnetic susceptibilities of guest-saturated samples with a 10 : 0 H_2_O–CH_3_OH ratio in intact (c) and partially guest-depleted (d) states were measured using the same method. (e) Local magnetic susceptibilities of the four guest-saturated samples, highlighting the 2nd-step spin transition with slow spin equilibrium (partial magnifications of (a–d)). Plots of temperature-scan rate *versus* residual HS population (*γ*_HS_) value for guest-saturated samples with 10 : 0 (f) and 8 : 2 (g) H_2_O–CH_3_OH ratios starting with different ambient conditions.

To further elucidate the high sensitivity of slow spin equilibrium to guest molecules, a comprehensive analysis was performed by varying the scan rate (10, 5, 2, 1, 0.5, 0.25, and 0.1 K min^−1^) and by *in situ* modulation of guest-saturated states (intact and partially guest-depleted state at 8 : 2 and 10 : 0 H_2_O–CH_3_OH ratios) using the semi-sealed method (Fig. 3 and S25–32). The guest-saturated samples with 8 : 2 and 10 : 0 H_2_O–CH_3_OH ratios were loaded at 250 K to preserve their intact states, while the corresponding partially guest-depleted states were obtained by *in situ* heating to 300 K and holding at this temperature for 10 min. The four types of guest-saturated samples exhibit two-step incomplete SCO properties at all scan rates, while the completeness of the 2nd-step spin transition is significantly affected by varying the scan rate. For the guest-saturated sample with an initial 10 : 0 H_2_O–CH_3_OH ratio, the minimum *γ*_HS_ values of the intact state range from 0.412 at 10 K min^−1^ (max. scan rate) to 0.159 at 0.1 K min^−1^ (min. scan rate), and from 0.224 at 10 K min^−1^ to 0.104 at 0.1 K min^−1^ for the partially guest-depleted state (Fig. 3c and d). In comparison, the min. *γ*_HS_ values of the intact and partially guest-depleted states of guest-saturated samples with an 8 : 2 H_2_O–CH_3_OH ratio are higher than those of the two states of the sample with a 10 : 0 H_2_O–CH_3_OH ratio at each scan rate (Fig. 3a–d). Specifically, the min. *γ*_HS_ values range from 0.469 at 10 K min^−1^ to 0.194 at 0.1 K min^−1^ for the intact state and from 0.329 at 10 K min^−1^ to 0.157 at 0.1 K min^−1^ for the partially guest-depleted state. The partially guest-depleted state exhibits greater SCO completeness at high scan rates compared to the intact state with the same initial H_2_O–CH_3_OH ratio, showing an increase of 18.8% at 10 K min^−1^ for the 10 : 0 H_2_O–CH_3_OH ratio and 14% at 10 K min^−1^ for the 8 : 2 H_2_O–CH_3_OH ratio (Fig. 3e). As the scan rate decreases, the gap between the two states diminishes, resulting in an increase in SCO completeness at 0.1 K min^−1^ by 5.5% for the 10 : 0 H_2_O–CH_3_OH ratio and 3.7% for the 8 : 2 H_2_O–CH_3_OH ratio. The partial loss of guest molecules in guest-saturated samples with either an 8 : 2 or 10 : 0 H_2_O–CH_3_OH ratio results in a slight narrowing of the first-step SCO (Fe1) thermal hysteresis loops, while the hysteresis widths remain stable and largely unaffected by scan rate, which is indicative of the robust reversibility (Fig. S26, S28, S30 and S32). The slow spin equilibrium of 2nd-step SCO (Fe_2_) is shown to be influenced by the manipulation of guest molecules, exhibiting regular behavior under the influence of guest load. In the partially guest-depleted state, the slow spin equilibrium of Fe_2_ is more easily overcome by scan rate than in the corresponding intact state, showing a larger difference at higher scan rates and a smaller difference at lower scan rates between the two states. For each guest-saturated sample, high scan rates (10 and 5 K min^−1^) have a minimal effect on the completeness of the 2nd-step spin transition. In contrast, medium scan rates (2, 1, and 0.5 K min^−1^) amplify the effect, a low scan rate (0.25 K min^−1^) has a minor effect, and an ultra-low scan rate (0.1 K min^−1^) exhibits an enlarged effect (Fig. 3f and g).

**Fig. 4:**
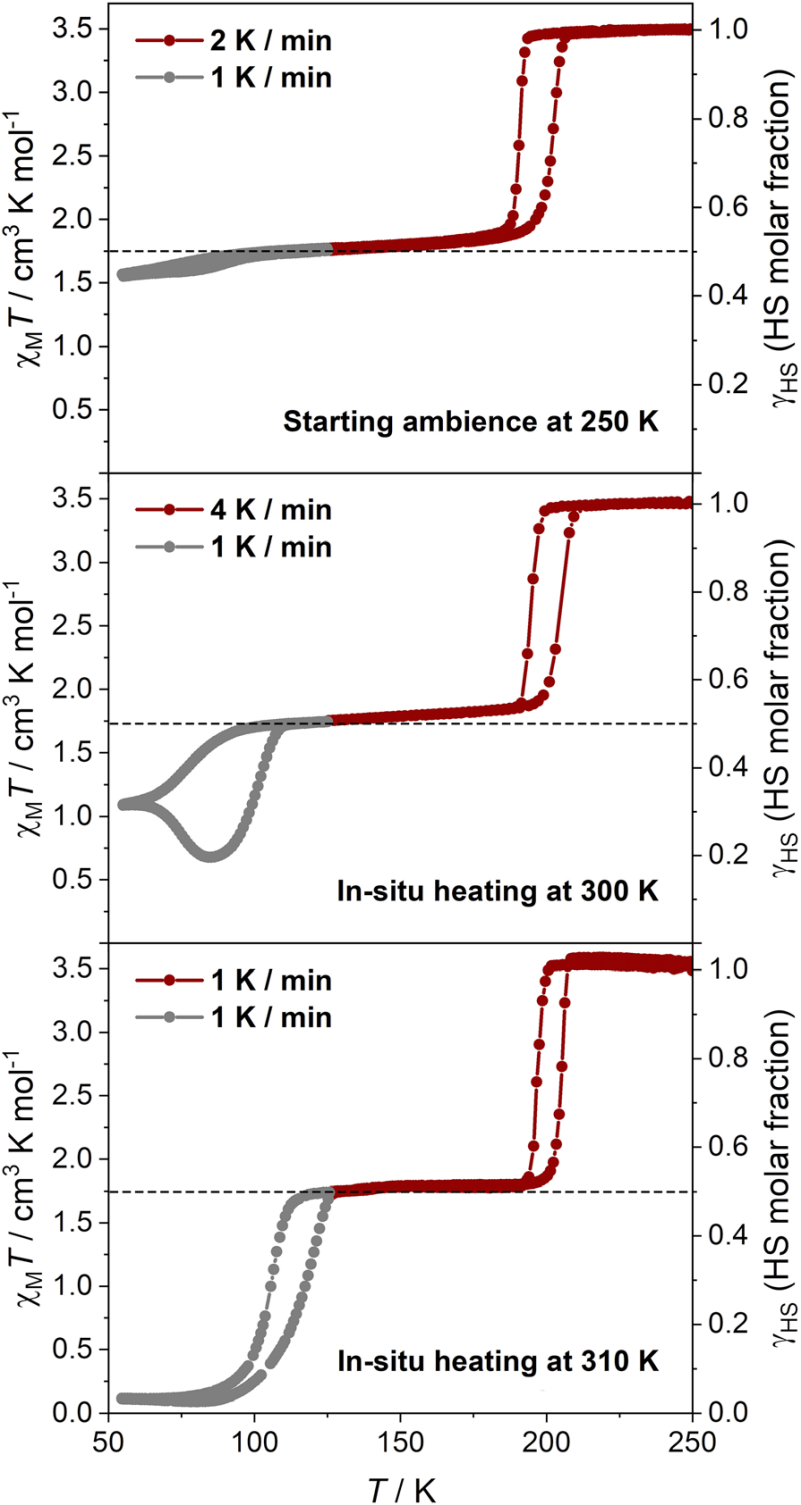
Temperature-dependent χ_M_*T* and *γ*_HS_ values of a guest-saturated sample with a 2 : 8 H_2_O–CH_3_OH ratio subjected to various temperature cycles during *in situ* gradual desolvation. A fresh sample was placed at an ambient temperature of 250 K before initiating the first cooling–warming cycle (top). The sample was then warmed to 300 K and held for 10 min, followed by cooling to 250 K for measurement in the second cooling–warming cycle (middle). Subsequently, the sample was warmed up to 310 K, held for 10 min, and cooled to 250 K for the third cooling–warming cycle (bottom). The dotted lines represent the HS_0.5_ Fe^II^ fraction showing the plateau of SCO behavior.

To thoroughly investigate how the number of guest molecules influences the SCO properties, magnetic susceptibilities of a guest-saturated sample with a 2 : 8 H_2_O–CH_3_OH ratio were measured after subjecting the sample to various temperature cycles during *in situ* gradual desolvation (Fig. 4 and S33–S36). When loaded at 250 K to maintain the intact state, the slow spin equilibrium of Fe_2_ was noted to be difficult to thermally overcome at 1 K min^−1^ (Fig. 4a). *In situ* warming to 300 K (held for 10 min) significantly enhanced the spin transition completeness of Fe_2_ in the partially guest-depleted state, with the transition unexpectedly dropping from 62 K and reaching a min. *γ*_HS_ value of 0.20 at 85 K (Fig. 4b and S33). This dynamic phenomenon is attributed to a temperature-induced excited spin-state trapping (TIESST) effect or slow spin equilibrium. Further *in situ* heating to 310 K (held for 10 min) caused the additional loss of guest molecules, allowing the spin equilibrium of Fe_2_ to be rapidly achieved, with the min. *γ*_HS_ value ultimately reaching 0.03 in this cooling–warming cycle (Fig. 4c). This indicates two-step complete SCO behavior, suggesting that the slow spin equilibrium of SCO has entirely disappeared. Subsequently, the sample was heated to 395 K to ensure that most guest molecules were lost, leading to two-step SCO without a plateau, corresponding to a single-crystal-to-single-crystal transformation, as described in previous studies (Fig. S34).^58^ The high-temperature-treated samples were rehydrated by immersion in water, and the magnetic susceptibilities of the rehydrated guest-saturated samples were measured using the fully sealed method (Fig. S35 and S36). The rehydrated samples recovered incomplete two-step SCO behavior with slow spin equilibrium. In comparison to the guest-saturated samples (a 10 : 0 H_2_O–CH_3_OH ratio) before thermal treatment, the temperature-dependent *γ*_HS_ values of the rehydrated samples showed that the residual HS population after the 2nd-step spin transition was slightly lower than that of the initial state at high scan rates (Δ*γ*_HS_ = 0.026 at 10 K min^−1^, 0.03 at 5 K min^−1^, and 0.015 at 2 K min^−1^), converging at 1 K min^−1^, and this is marginally higher at low scan rates (Δ*γ*_HS_ = −0.03 at 0.5 K min^−1^, −0.027 at 0.25 K min^−1^, and −0.027 at 0.1 K min^−1^). These differences are minor and probably result from particle size changes caused by repeated thermal cycling. Hence, as the guest molecules are gradually lost, the slow equilibrium in guest-saturated samples shifts—from being thermally difficult to overcome to becoming easily thermally accessible, and eventually transitioning fully, indicative of a fast spin equilibrium. Regulating SCO with slow spin equilibrium through guest molecule loss is more challenging and has a greater impact on the elastic frustration of the framework than simply altering the type and ratio of the immersion solvent.

## Conclusions

In conclusion, a strategy for guest-manipulated slow spin equilibrium during a two-step SCO process is proposed to achieve the bidirectional modulation of the untouched region between one-step incomplete (HS_0.5_LS_0.5_ ↔ HS) and two-step complete (LS ↔ HS_0.5_LS_0.5_ ↔ HS) SCO behaviors in Hofmann-type MOFs. The SCO properties of a framework stuffed with H_2_O *versus* D_2_O states when examining scan-rate-dependent slow spin equilibrium exclude the effects of the size of the guest molecule. The ranking of H_2_O, D_2_O, and CH_3_OH guest molecules regarding the degree of SCO completeness is summarized, while the PXRD data for different guest-saturated states indicate the contraction/expansion of the framework. This also impacts the elastic frustration of the framework structure, which is consistent with the effect on the spin equilibrium of Fe_2_. The SC-XRD data for 1·3H_2_O·3/2CH_3_OH (the guest-saturated sample with a 5 : 5 H_2_O–CH_3_OH ratio) reveal that CH_3_OH molecules preferentially replace H_2_O molecules in the central part of the pore cavity while the overall framework does not change significantly. Even though the potential mechanisms controlling these slow spin equilibria have not been fully established, it is evident that guest molecules can achieve the specific regulation of slow spin equilibrium in guest-saturated states through host–guest interactions. This provides novel insights and methods for designing new SCO material systems and for rationally modeling theoretical calculations of SCO textures.

## Author contributions

J.-P. Xue proposed the idea and designed the research. J.-P. Xue, Y. Chai, and Y.-T. Yang performed syntheses and measurements. J.-P. Xue and Y. Chai performed the magnetic and SC-XRD measurements. Y.-T. Yang performed the PXRD and micro-Raman measurements. J.-P. Xue supervised the research. J.-P. Xue and Y. Chai wrote the manuscript. All authors discussed the results and commented on the manuscript.

## Conflicts of interest

There are no conflicts to declare.

## Supplementary Material

SC-OLF-D5SC01202C-s001

SC-OLF-D5SC01202C-s002

## Data Availability

The data supporting this article have been included as part of the SI. The crystallographic data for structures reported in this study have been deposited at the Cambridge Crystallographic Data Centre (CCDC) under the deposition numbers CCDC 2357594 (the guest-saturated state 1·3H_2_O·3/2CH_3_OH at 293 K), 2357595 (the guest-saturated state 1·3H_2_O·3/2CH_3_OH at 190 K), 2350745 (the guest-saturated state 1·3H_2_O·3/2CH_3_OH at 150 K), 2350741 (the guest-saturated state 1·3H_2_O·3/2CH_3_OH at 130 K), 2357603 (the guest-saturated state 1·3H_2_O·3/2CH_3_OH at 120 K), and 2357613 (the guest-saturated state 1·3H_2_O·3/2CH_3_OH at 85 K). CCDC 2350741, 2350745, 2357594, 2357595, 2357603 and 2357613 contain the supplementary crystallographic data for this paper.^65–70^ Supplementary information is available, including detailed synthetic methods, experimental procedures, micro-Raman measurements , and additional figures. See DOI: https://doi.org/10.1039/d5sc01202c.
