# Supplementary material for: Entanglement of spin transition and structural adaptability: manipulating the slow spin equilibrium by the guest-mediated fine-tuning of elastic frustration
Source: Chem Sci. 2025 Jul 21;16(34):15555–63. doi: 10.1039/d5sc01202c (PMC12312210; doi:10.1039/d5sc01202c)
Supplement: SC-016-D5SC01202C-s001 [file SC-016-D5SC01202C-s001.pdf]

Supplementary Information (SI) for Chemical Science.
This journal is © The Royal Society of Chemistry 2025

Supplementary Information for:

Entanglement of Spin Transition and Structural Adaptability: Manipulating the Slow Spin Equilibrium by Guest-Mediated Fine-Tuning Elastic Frustration

Yuqiao Chai,[a] Yu-Ting Yang,[a,b] and Jin-Peng Xue[b,c]*

[a] School of Material and Chemical Engineering, Ningbo University of Technology, Ningbo, Zhejiang, 315211, China.

[b] School of Materials Science and Chemical Engineering, Ningbo University, Ningbo, 315211, China

[c] Jiangxi Provincial Key Laboratory of Functional Crystalline Materials Chemistry, Jiangxi University of Science and Technology, Ganzhou 341000, Jiangxi, China.

*Correspondence to: xuejinpeng@nbu.edu.cn (J.-P. Xue.).

General Experimental Section:

Synthesis of ligand (1*E*,2*E*)-3-phenyl-N-(4H-1,2,4-triazol-4-yl)prop-2-en-1-imine (prentrz). The ligand prentrz was synthesized by a modified literature procedure.[1,2]

Synthesis of [Fe^{II}(prentrz)$_2$$Pd^{II}(CN)_4$]·guest (1·guest) and guest exchange. The pristine crystals of **1·guest** were synthesized by the method of slow diffusion reported previously.[2] The samples in the guest-saturated state with H_2O-CH_3OH (10:0, 8:2, 5:5, 2:8), H_2O-D_2O (5:5), and D_2O-CH_3OH (10:0, 8:2) ratios were obtained by guest exchange through immersion of the as-synthesized samples in respectively pure mixture solutions (10 mL) for 3 times.

Single-Crystal X-ray Diffraction (SC-XRD) Measurements. The crystal data for the guest-saturated state **1**·3H_2O·3/2CH_3OH were recorded at 293, 190, 150, 130, 120, and 85 K, respectively, with a Bruker/ARINAX MD2 diffractometer equipped with a MarCCD-300 detector (λ = 0.77484 Å) at the BL17B beamline station of the Shanghai Synchrotron Radiation Facility (SSRF). A total of 360 frames were collected using ω-scans with the 1° oscillation angle, spanning from 0 to 360°, an exposure time of 0.50 s per frame, and a detector distance of 90 mm. The BlueIce software was used to collect data. The dataset was collected on the beamline configured with a single-axis goniometer, which enabled phi-scans. However, the orientation of the small-molecule crystal and its low symmetry presented challenges in capturing 100% of reflections within this setup. Unit cell parameters were refined and data reduction was carried out using the HKL3000 software.[3]

All the structures were determined using direct methods and subsequently refined through full-matrix least-squares techniques on F^2 using the SHELX program.[4] Non-hydrogen atoms underwent anisotropic refinement, whereas hydrogen atoms were placed in geometrically idealized positions and refined with isotropic displacement parameters.

Powder X-ray diffraction (PXRD) Measurements. The PXRD data were recorded on a Bruker D8 ADVANCE diffractometer with Cu Kα radiation at room temperature. The samples immersed in solutions with H_2O-CH_3OH (10:0, 8:2, 5:5), H_2O-D_2O (5:5), and D_2O-CH_3OH (10:0, 8:2) ratios were

loaded on the glass sample table with a circular groove (diameter ~2 cm, thickness ~1 mm). After flattening and compacting the samples, two drops of the solution with the corresponding ratio were added to the sample surface, and then the glass sample table including samples was coated with a PE film to keep the guest-overload state during the measurement.

Magnetic Measurements. The measurements of variable-temperature magnetic susceptibilities were performed on a Quantum Design MPMS XL-7 magnetometer working in the range of 2–400 K with 10, 5, 2, 1, 0.5, 0.25, and 0.1 K min^{-1} sweeping rate under the 5000 Oe magnetic field.

The magnetic test samples are divided into two production methods according to the full-sealed and semi-sealed conditions as follows:

The full-sealed method involved high-temperature sintering of a quartz tube containing guest-exchange samples. The as-synthesized samples were placed at the bottom of the quartz tube, and guest exchange was performed as the same method described above. After the exchange, the excess solutions were thoroughly removed. The sample was then vacuum-sealed while being frozen in liquid nitrogen. During this process, a handheld gas torch was used to sinter and seal the quartz tube approximately 3 cm above the samples. The sealed quartz tube containing the samples was subsequently fixed for magnetic testing.

The semi-sealed method involved wrapping the guest-exchange samples with a PE film. The samples were placed in the center of the PE film (3×3 cm^2), followed by the addition of two drops of the corresponding pure mixture solutions, then quickly wrapped and folded. Absorbent cotton was used to soak up the excess solution around the sample, which was subsequently wrapped with an additional layer of PE film. The samples were loaded into the SQUID chamber at 250 K.

Assignation of the guest molecules types in the guest-saturated state. Single-crystal X-ray diffraction (SC-XRD) data are utilized to determine the number and type of solvent molecules within the channel-type pore. To eliminate this potential interference, SC-XRD data of the guest-saturated state (**1**·$3H_2O$·$3/2CH_3OH$) at 150 K is compared with previously published SC-XRD data of the stable

state in air (1·9/2H_2O) at 150 K (Figure S2). The Q peak in close proximity to the uncoordinated nitrogen atoms of the prentrz ligand is unambiguously assigned as a water molecule (O1).

The residual four Q peaks with heights of 2.5, 5.61, 6.14, and 7.7 were observed in the pore of the framework structure in **1**·9/2H_2O. These Q peaks, being relatively far from each other, can more reasonably be assigned as water molecules. The relatively high heights of Q peaks may be attributed to a network of hydrogen bonding interactions formed between water guest molecules.

For **1**·3H_2O·3/2CH_3OH, excluding the Q peak identified as O1, the pore is dominated by residual six Q peaks with heights of 2.36, 2.38, 2.87, 3.72, 5.16, and 5.94. The Q peaks located in the middle region of the pore are relatively close to each other, which can be recognized as methanol molecules, or water molecules by means of confinement operation. Given that the SC-XRD data of **1**·3H_2O·3/2CH_3OH are collected in the immersed mixed solution, the Q peaks here are preferred to be assigned as methanol molecules. The relatively low heights of the Q peaks may be due to the presence of methanol molecules disrupting the hydrogen bonding network originally formed by the water molecules, resulting in a weakening of the Q peak heights.

Micro-Raman spectroscopy. The micro-Raman spectra experiments were performed on a DXR3 Raman spectrometer equipped with an Olympus microscope. The excitation source was provided by an Argon ion laser with wavelength of 532 nm and output power of 6 mW (model Stellar-REN, Modu-Laser). The guest-saturated samples prepared with pure H_2O and D_2O placed in the groove of a custom-made quartz tray using a 20 × objective lens and covered with a cover glass. The tray was then sealed with polyethylene film, with a capillary column extending outside the apparatus to allow solvent injection by syringe. The spectra in the range 100−3500 cm^{-1} were collected with three spectral scans at 10 s accumulation time.

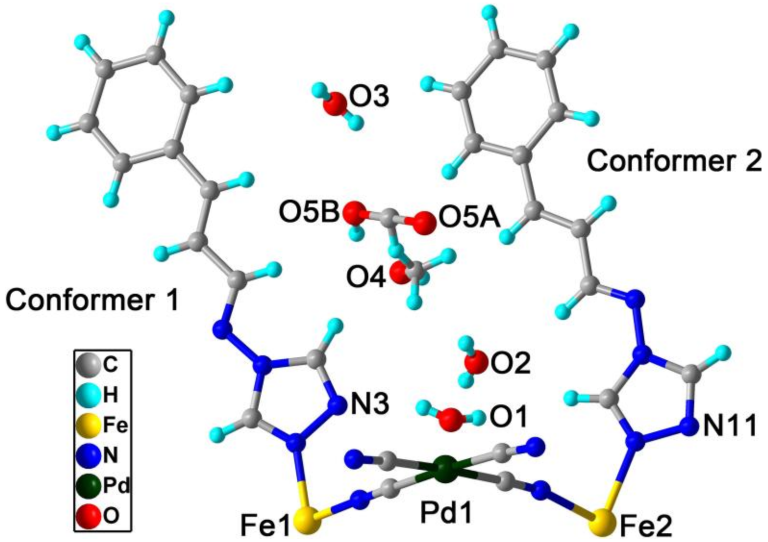

Figure S1. The asymmetric unit of **1**·$3H_2O$·$3/2CH_3OH$.

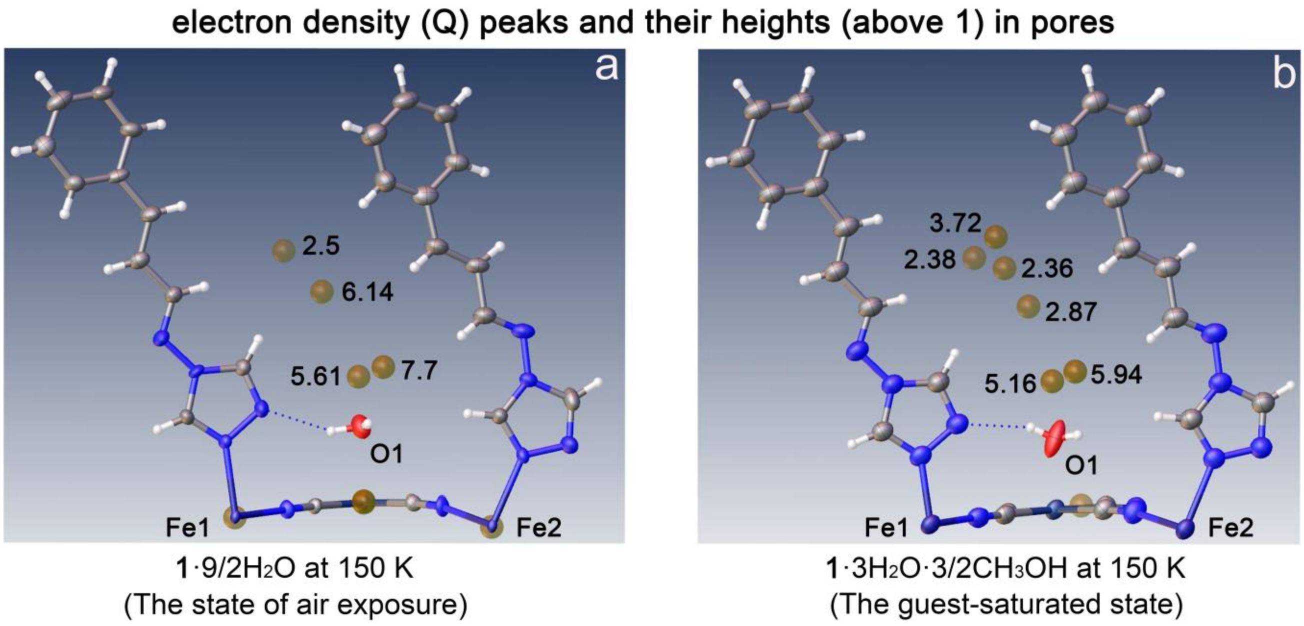

Figure S2. The asymmetric unit of the stable state in air (**1**·$9/2H_2O$, left) and the guest-saturated state (**1**·$3H_2O$·$3/2CH_3OH$, right) at 150 K. The Q peaks in the pore of the framework remain unassigned, except for a notable Q peak associated with O1, which has been identified as H_2O. The other Q peaks have not been assigned.

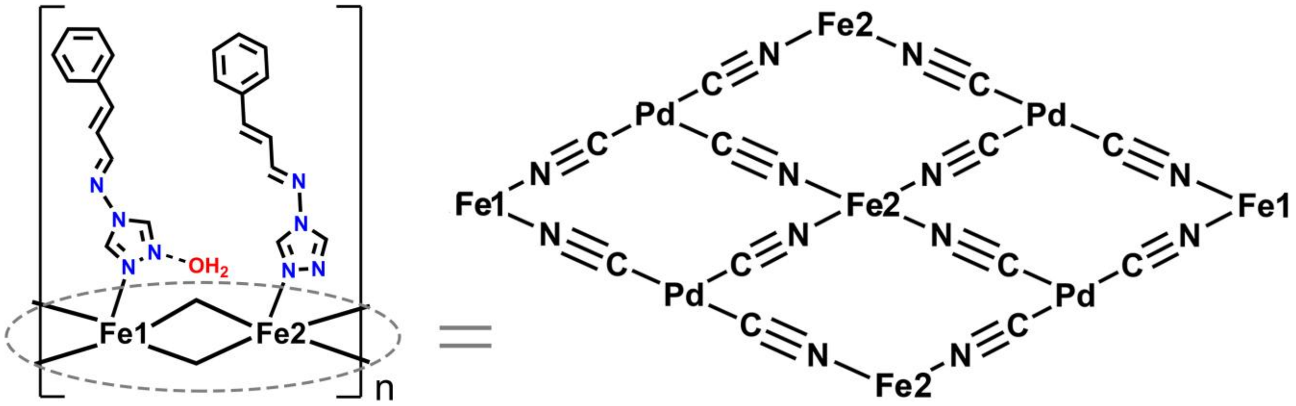

Figure S3. Building units of the title 2D coordination polymer.

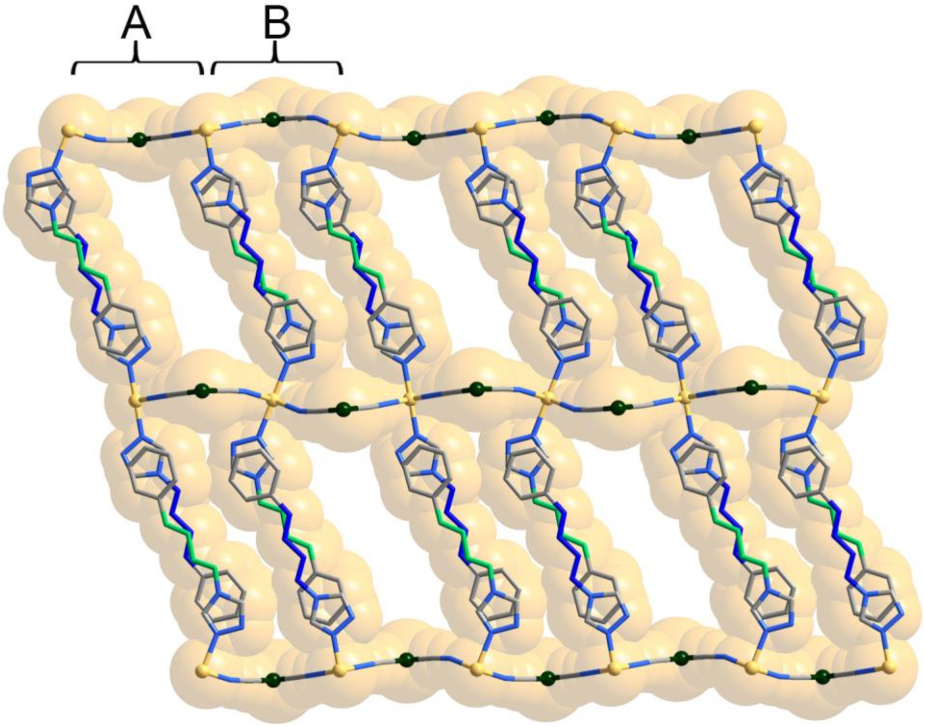

Figure S4. Pore structure and supramolecular packing of **1**·$3H_2O$·$3/2CH_3OH$.

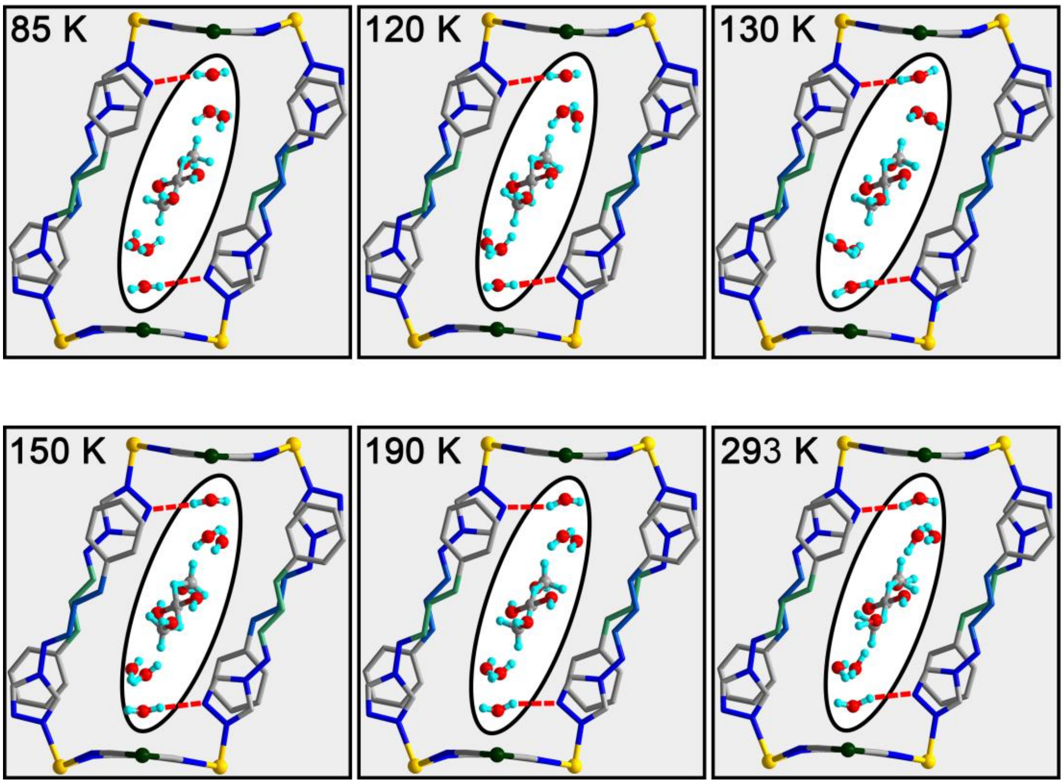

Figure S5. Distribution of H_2O and CH_3OH in **1**·$3H_2O$·$3/2CH_3OH$ at 85, 120, 130, 150, 190, and 293 K.

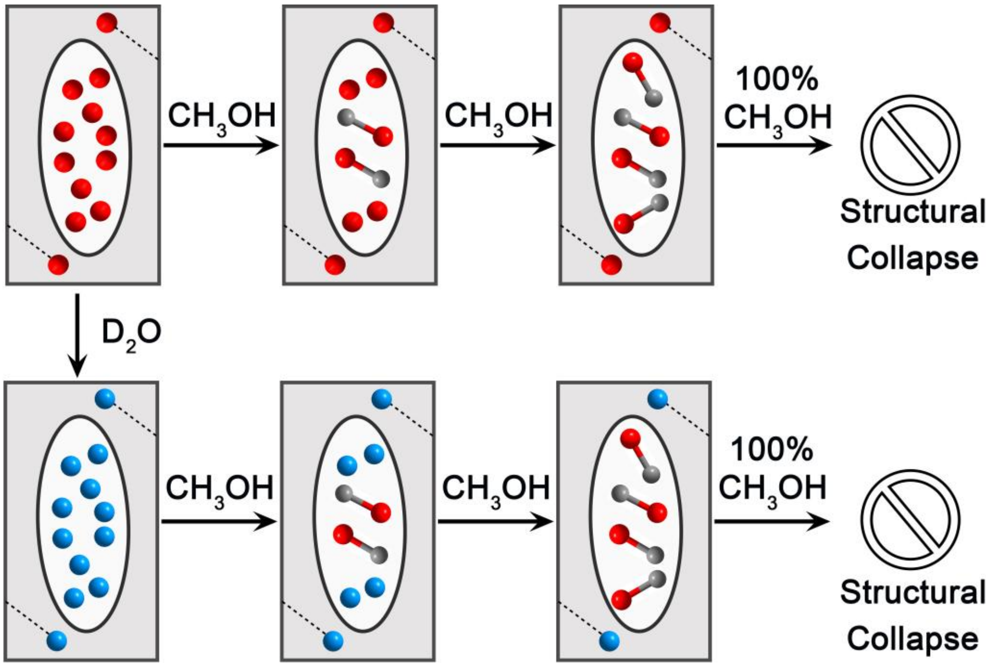

Figure S6. The hypothesized distribution of guest-saturated samples following exchange with CH_3OH, H_2O, and D_2O, and the inferred instability of the framework in pure CH_3OH.

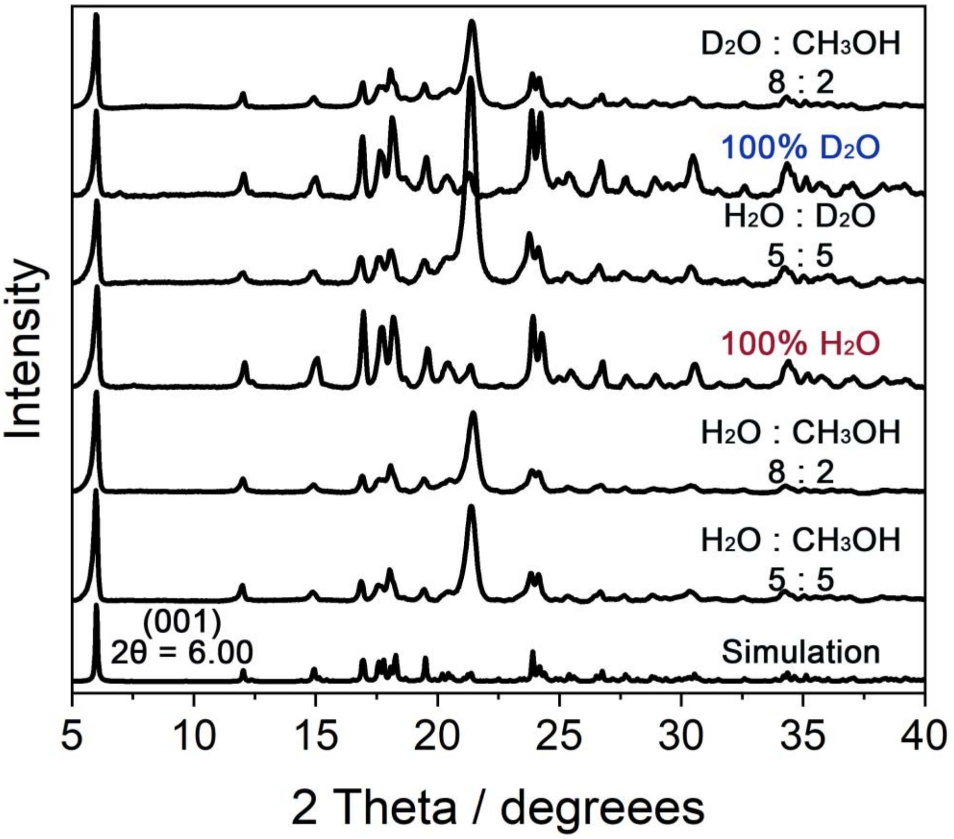

Figure S7. The full PXRD patterns of the guest-saturated samples during the guest-exchange process.

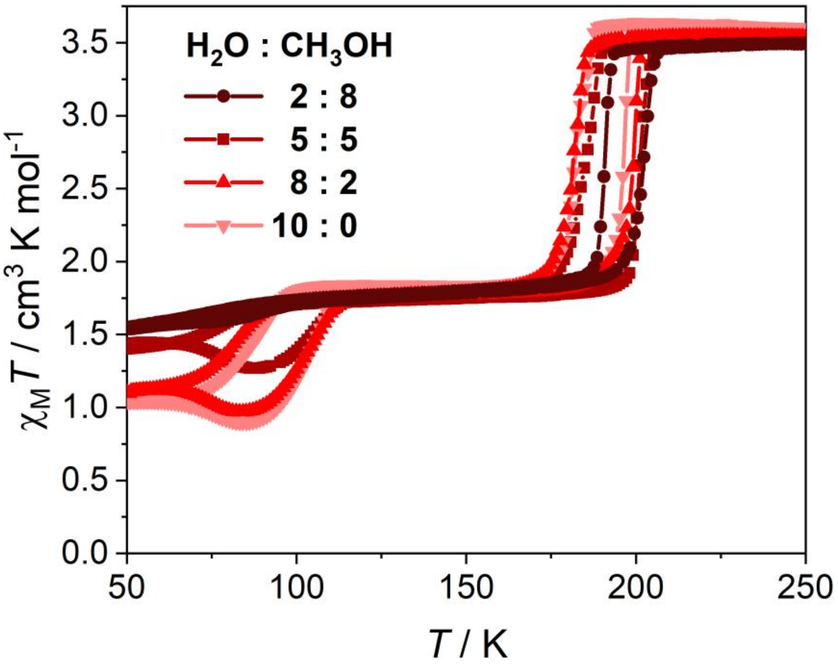

Figure S8. Temperature-dependent $\chi_M T$ values of the guest-saturated samples with various H_2O-CH_3OH ratios.

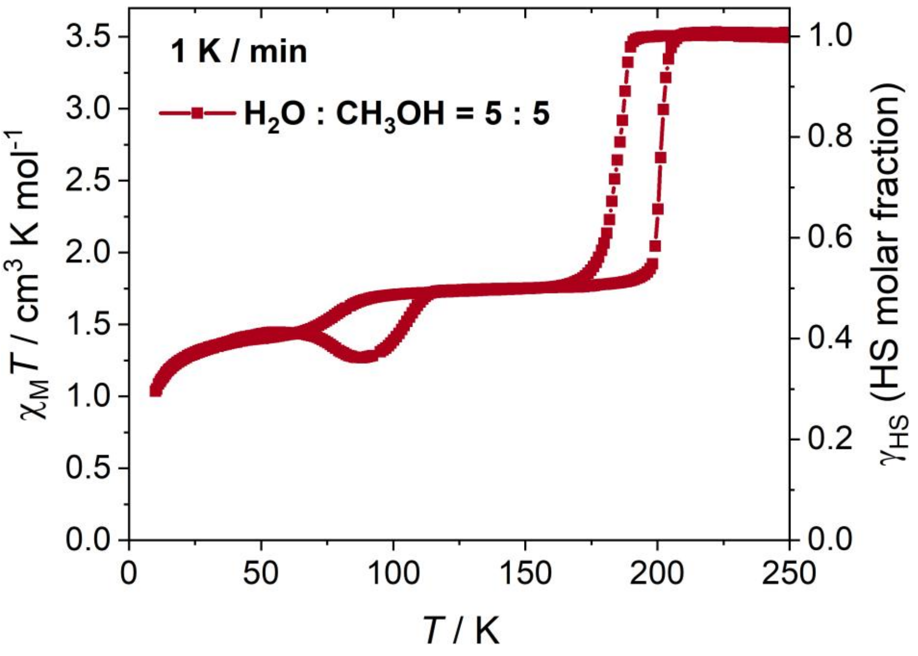

Figure S9. Temperature-dependent $\chi_M T$ values of the guest-saturated samples with 5:5 H_2O-CH_3OH ratio at 1 K min^{-1} (2 K to 250 K).

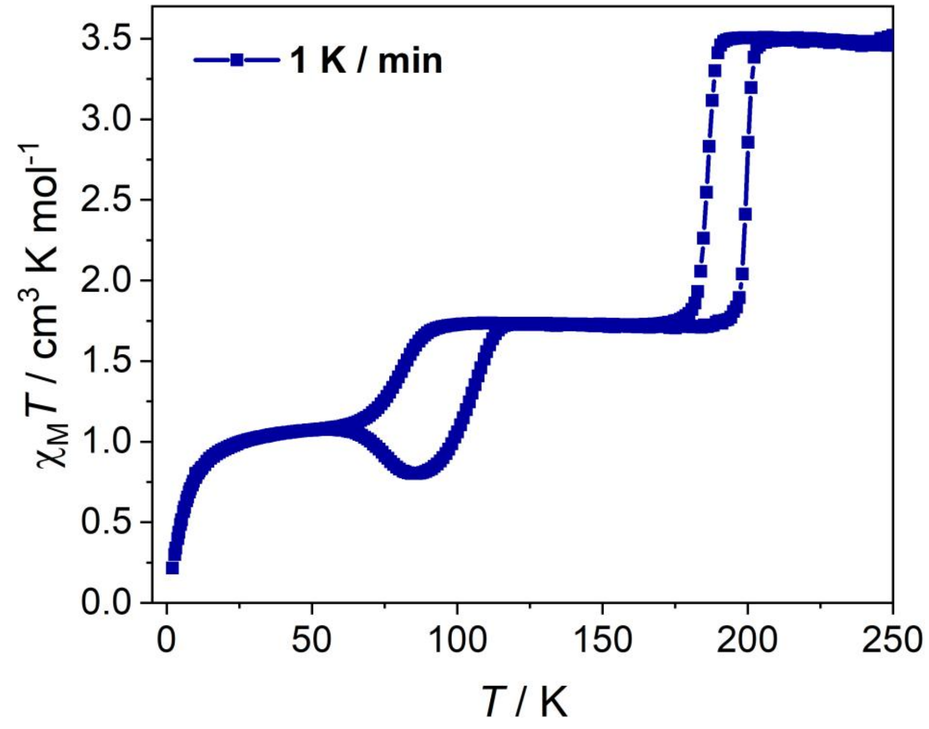

Figure S10. Temperature-dependent $\chi_M T$ values of the guest-saturated samples with 10:0 H_2O-CH_3OH ratio at 1 K min^{-1} (2 K to 250 K).

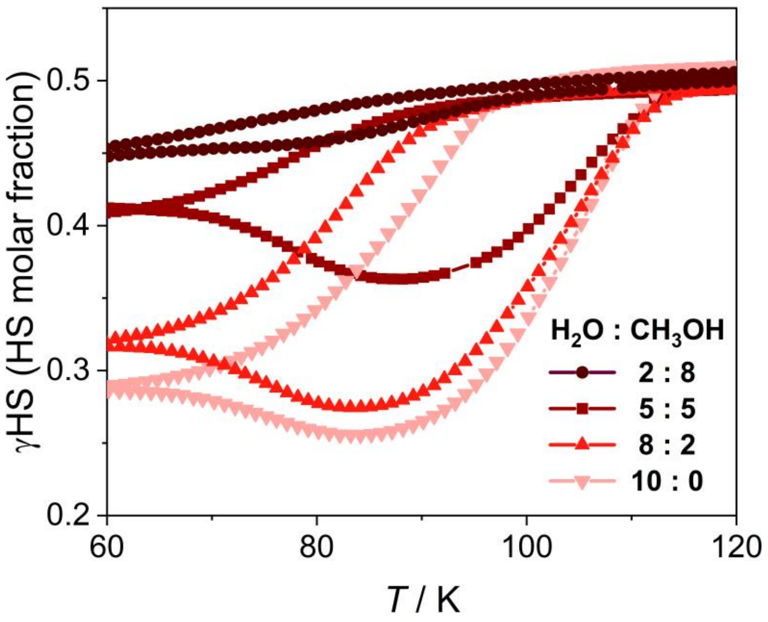

Figure S11. The enlarged view of the $\chi_M T$ of the guest-saturated samples with various H_2O-CH_3OH ratios product in the interval of 60−120 K.

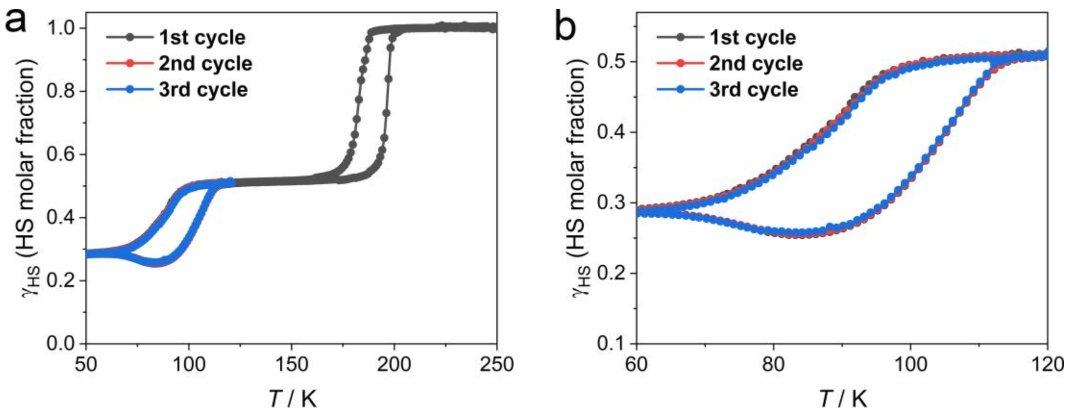

Figure S12. The reversibility of the spin equilibrium associated with the second-step spin transition. a, Temperature-dependent HS Fe^{II} molar fraction (γ_{HS}) values of the guest-saturated sample with 10:0 H_2O-CH_3OH ratio at 1 K min^{-1} (50 K to 250 K). The fresh sample was first measured in both cooling and warming modes between 50 and 250 K (first cycle). Subsequently, the sample was cooled to 120 K and subjected to another set of cooling and warming measurements between 50 and 120 K (second cycle). Afterward, the same thermal cycling procedure was performed once again as the third cycle. **b,** The enlarged view of the γ_{HS} values for the guest-saturated samples (H_2O-CH_3OH) in the temperature range of 60–120 K.

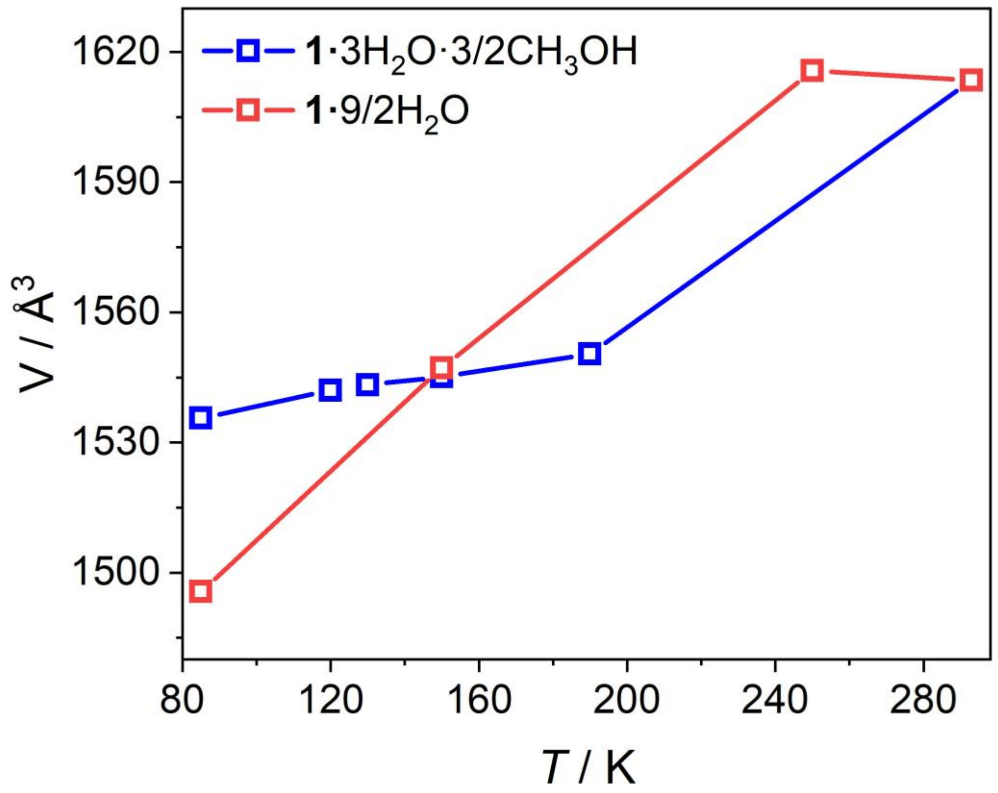

Figure S13. Temperature-dependent unit-cell volume of **1**·9/2H_2O and **1**·3H_2O·3/2CH_3OH.

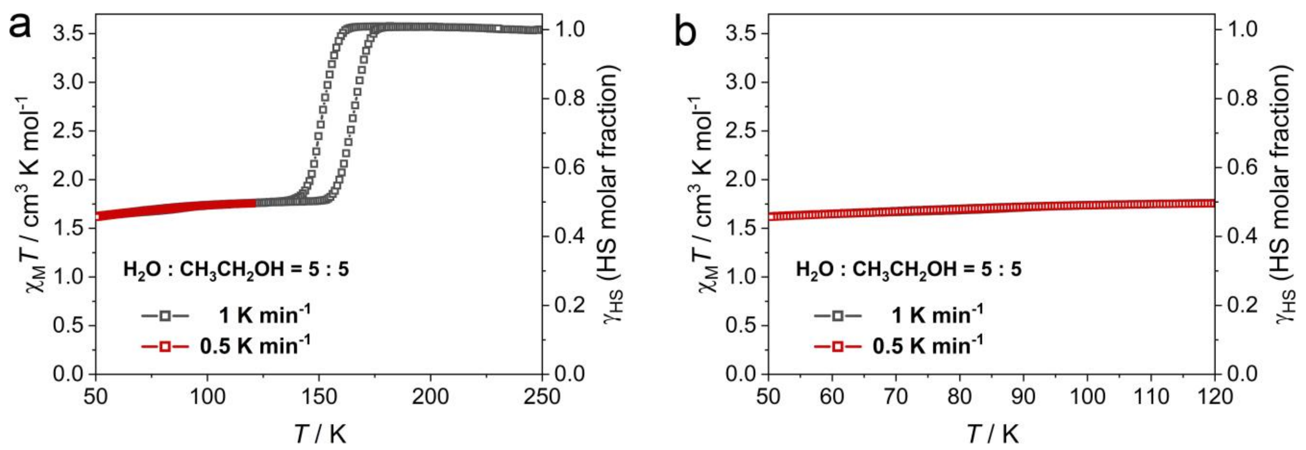

Figure S14. Temperature-dependent $\chi_M T$ values of the guest-saturated sample with 5:5 H_2O-CH_3CH_2OH ratio at 1 K min^{-1} (50 K to 250 K) and 0.5 K min^{-1} (50 K to 120 K), showing an incomplete one-step SCO without slow spin equilibrium.

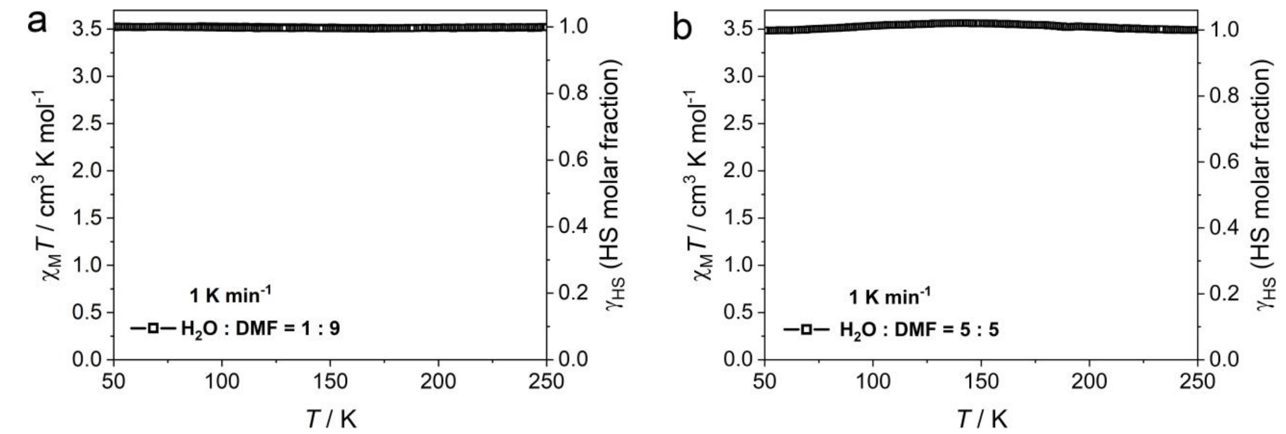

Figure S15. Temperature-dependent $\chi_M T$ values of the guest-saturated samples with 1:9 and 5:5 H_2O-DMF ratios at 1 K min^{-1} (50 K to 250 K), showing the HS state and the loss of SCO behavior.

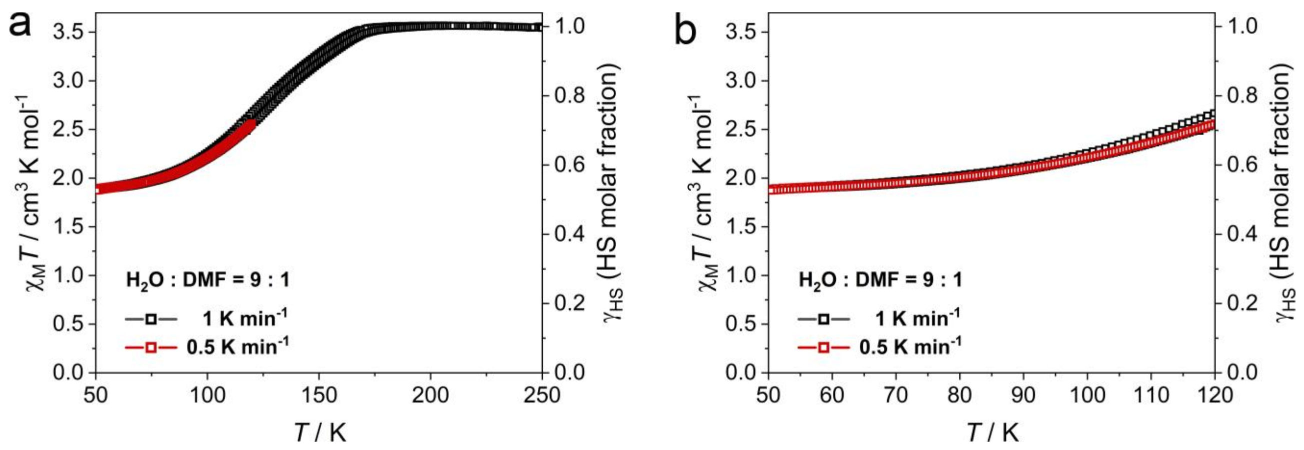

Figure S16. Temperature-dependent $\chi_M T$ values of the guest-saturated sample with 9:1 H_2O-DMF ratio at 1 K min^{-1} (50 K to 250 K) and 0.5 K min^{-1} (50 K to 120 K), showing an incomplete one-step SCO without slow spin equilibrium.

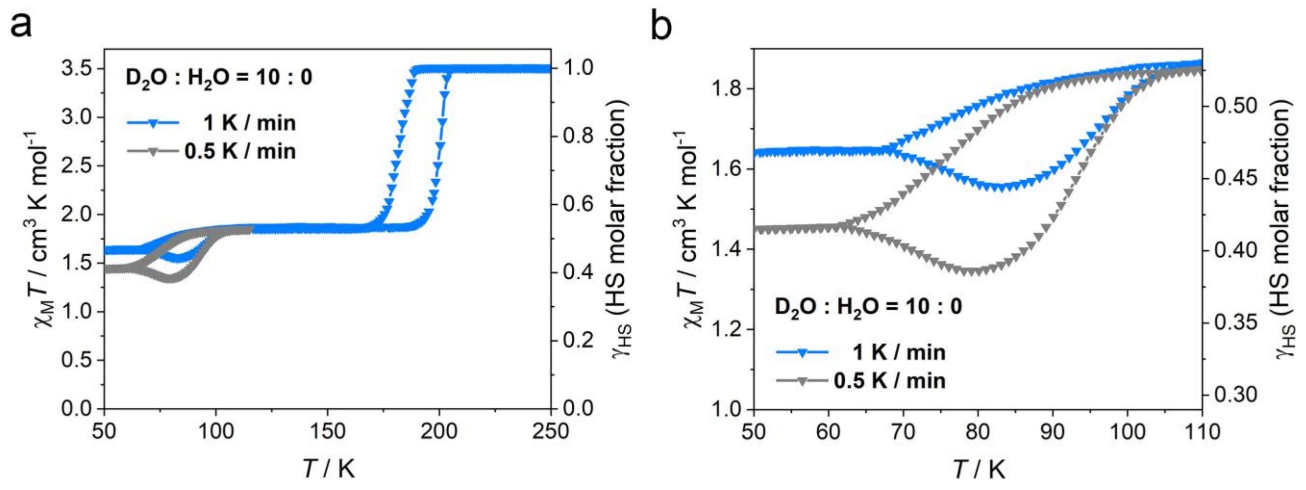

Figure S17. **a**, Temperature-dependent $\chi_M T$ values of the guest-saturated samples with 10:0 D_2O-CH_3OH ratio at 1 and 0.5 K min^{-1}. **b**, The enlarged view of the $\chi_M T$ product in the interval of 50−110 K.

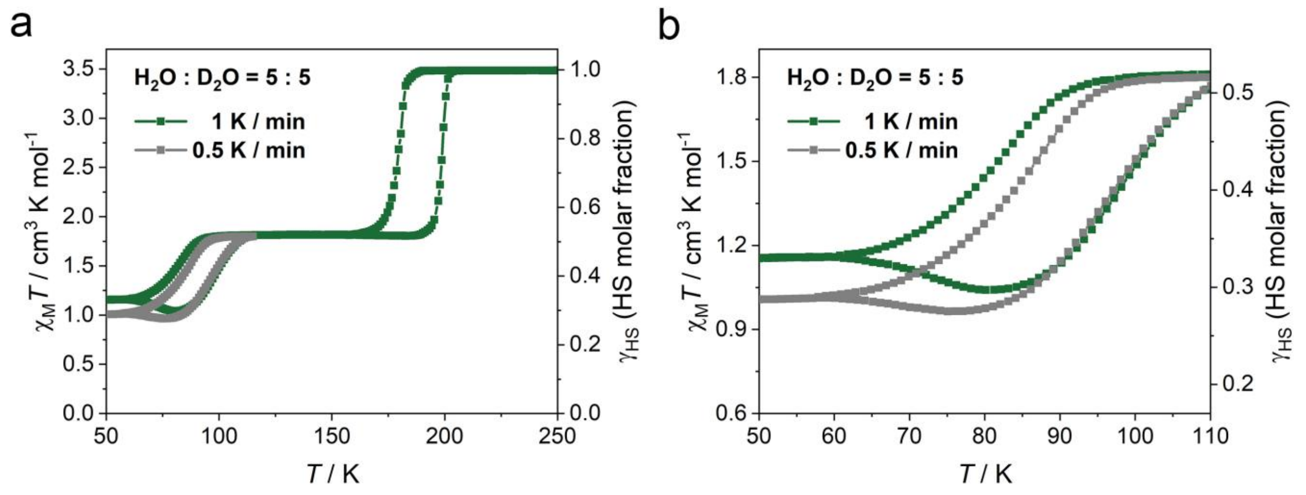

Figure S18. **a**, Temperature-dependent $\chi_M T$ values of the guest-saturated samples with 5:5 H_2O-D_2O ratio at 1 and 0.5 K min^{-1}. **b**, The enlarged view of the $\chi_M T$ product in the interval of 50−110 K.

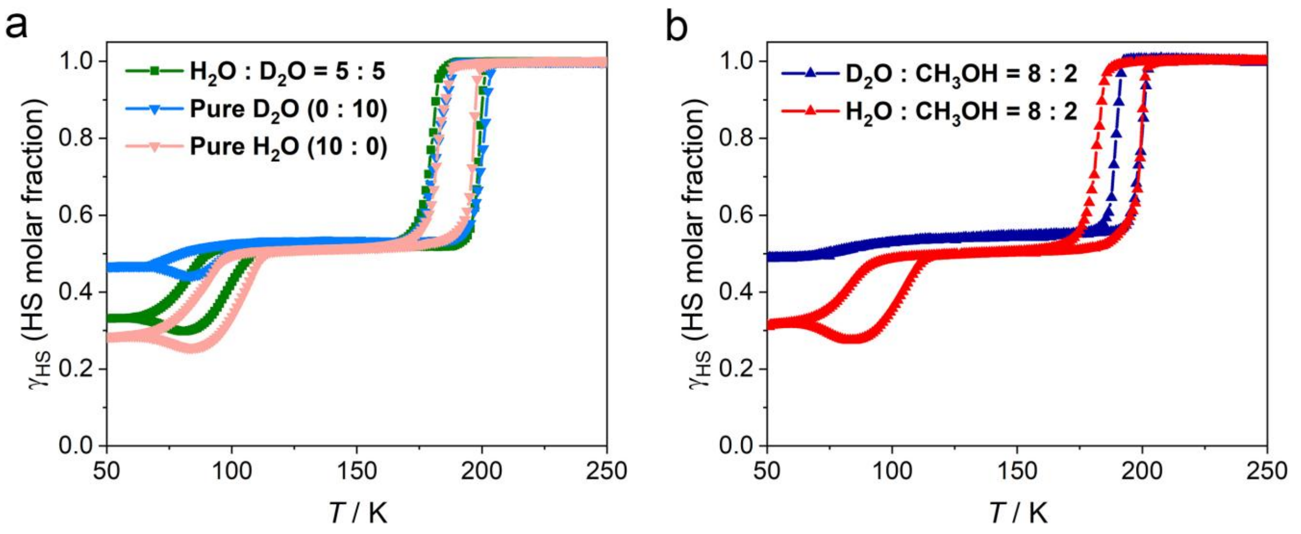

Figure S19. a, Temperature-dependent $\chi_M T$ values of the guest-saturated samples with pure D_2O, pure H_2O and 5:5 H_2O-D_2O ratio at 1 K min^{-1}. **b**, Temperature-dependent $\chi_M T$ values of the guest-saturated samples with 8:2 H_2O-CH_3OH and D_2O-CH_3OH ratios at 1 K min^{-1}.

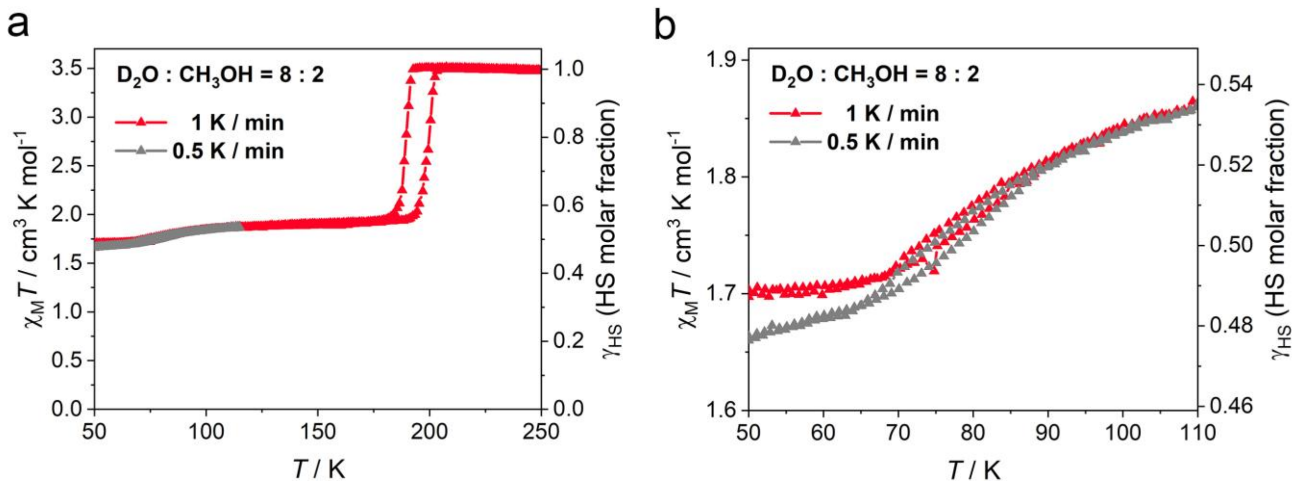

Figure S20. a, Temperature-dependent $\chi_M T$ values of the guest-saturated samples with 8:2 D_2O-CH_3OH ratio at 1 and 0.5 K min^{-1}. **b**, The enlarged view of the $\chi_M T$ product in the interval of 50−110 K.

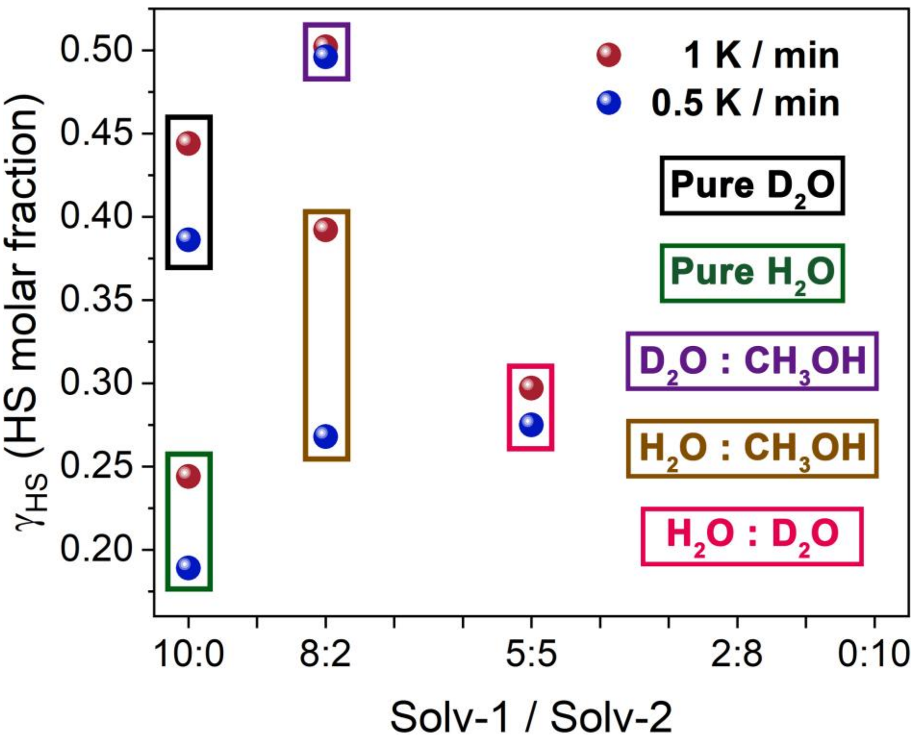

Figure S21. Plot of the guest dependence of the HS Fe^{II} molar fraction (γ_{HS}). The Solv-1/Solv-2 values on the *x*-axis represent the proportion of the mixed solvents described in the Figures S8-11 and S13-S16.

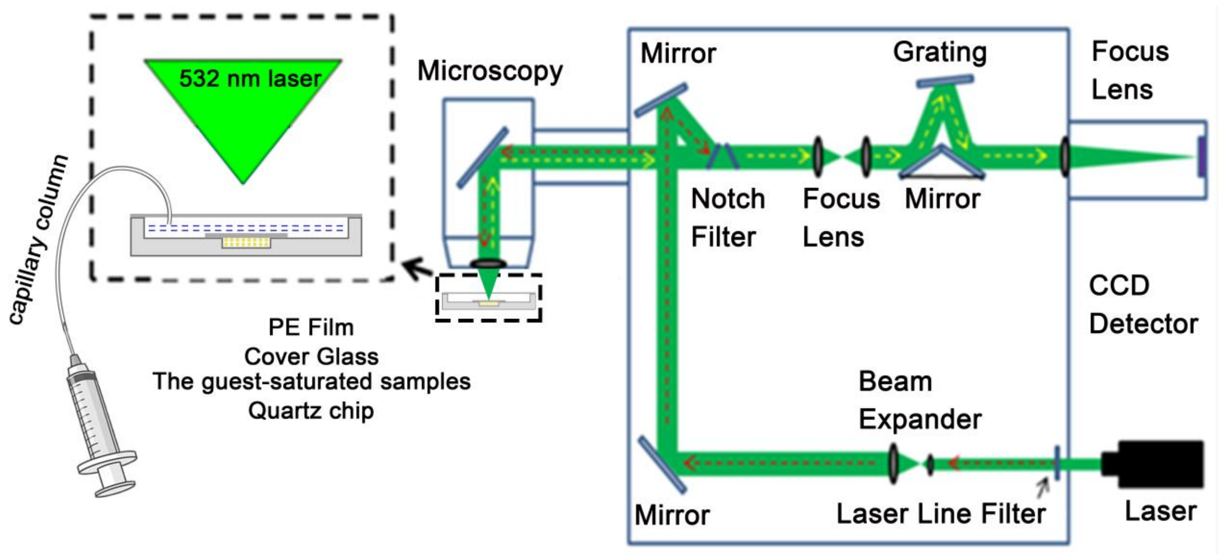

Figure S22. Experimental setup for Micro-Raman spectroscopy with different solvent ambience.

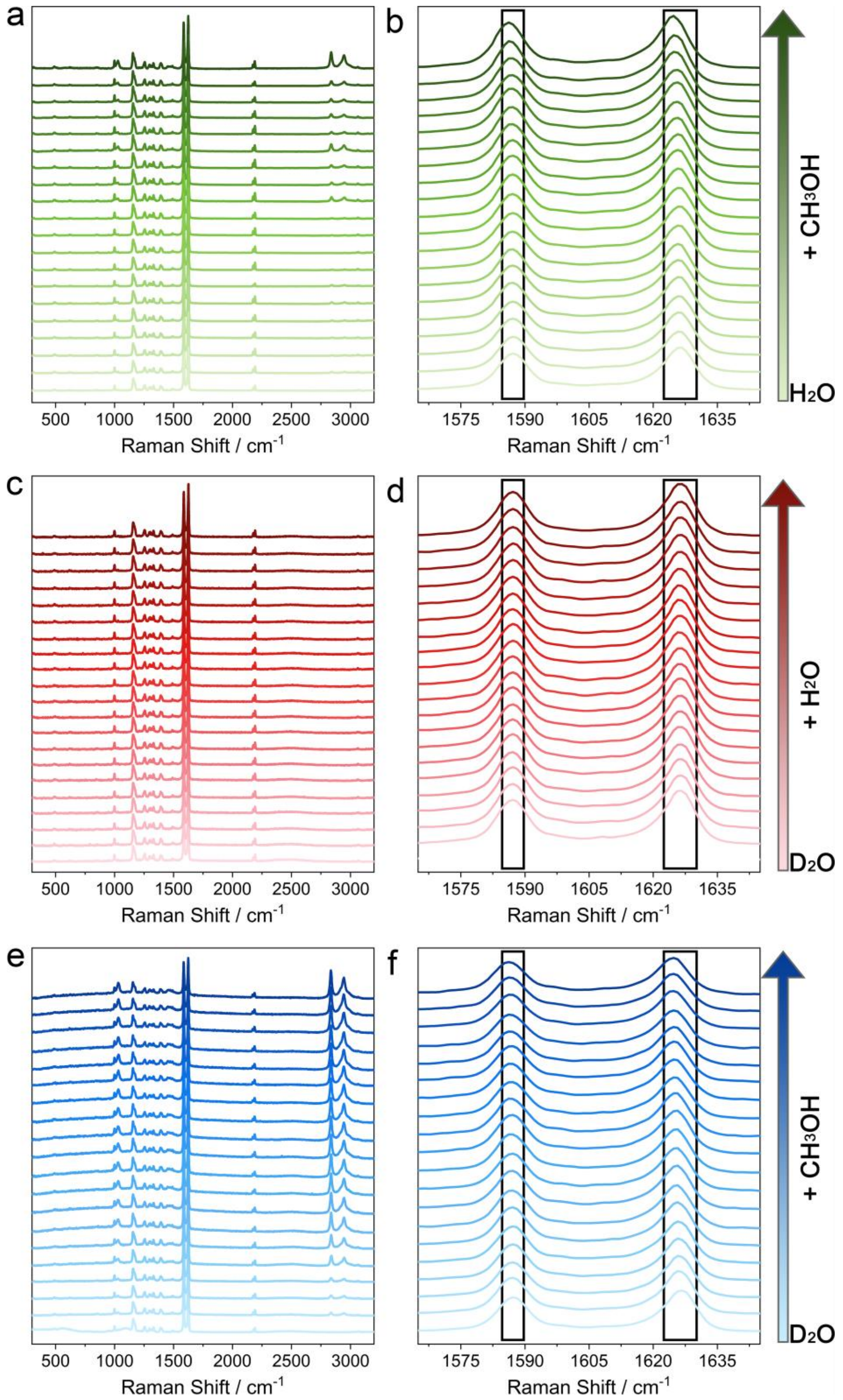

Figure S23. Time-resolved in situ micro-Raman spectra of the guest-saturated samples under different guest exchange conditions: **a, b** The guest-saturated sample with pure H_2O upon incremental addition of CH_3OH. **c, d** The guest-saturated sample with pure D_2O upon incremental addition of H_2O. **e, f** The guest-saturated sample with pure D_2O upon incremental addition of CH_3OH.

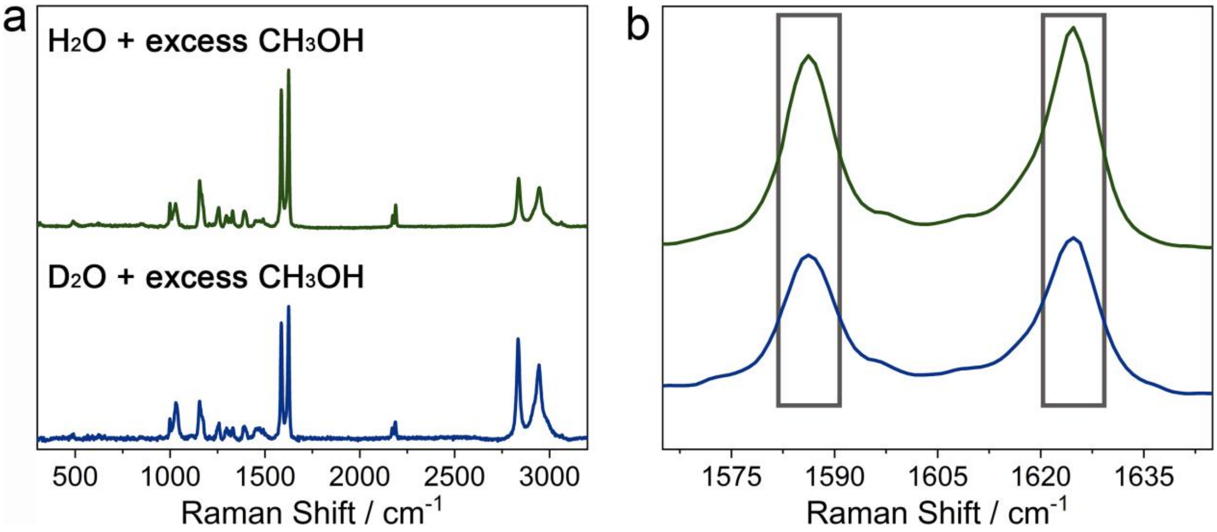

Figure S24. Micro-Raman spectra of the guest-saturated samples with pure H_2O and D_2O upon incremental addition of excess CH_3OH.

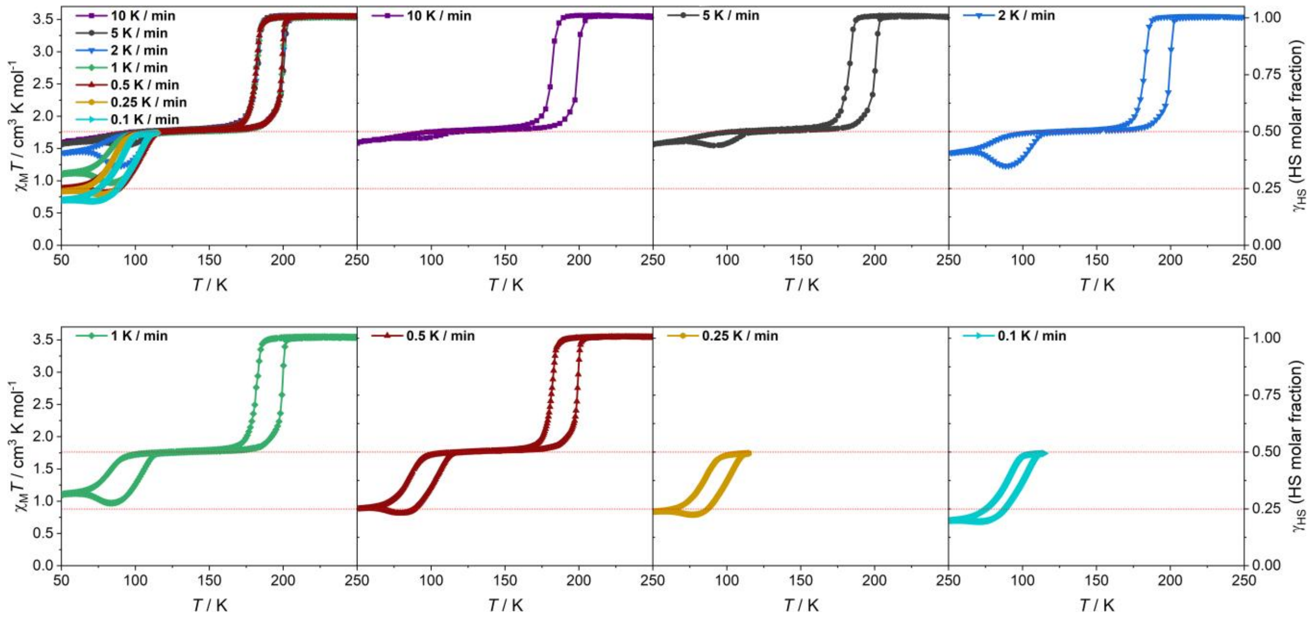

Figure S25. Temperature-dependent $\chi_M T$ values of the guest-saturated samples with 8:2 H_2O-CH_3OH ratio at an ambient temperature of 250 K.

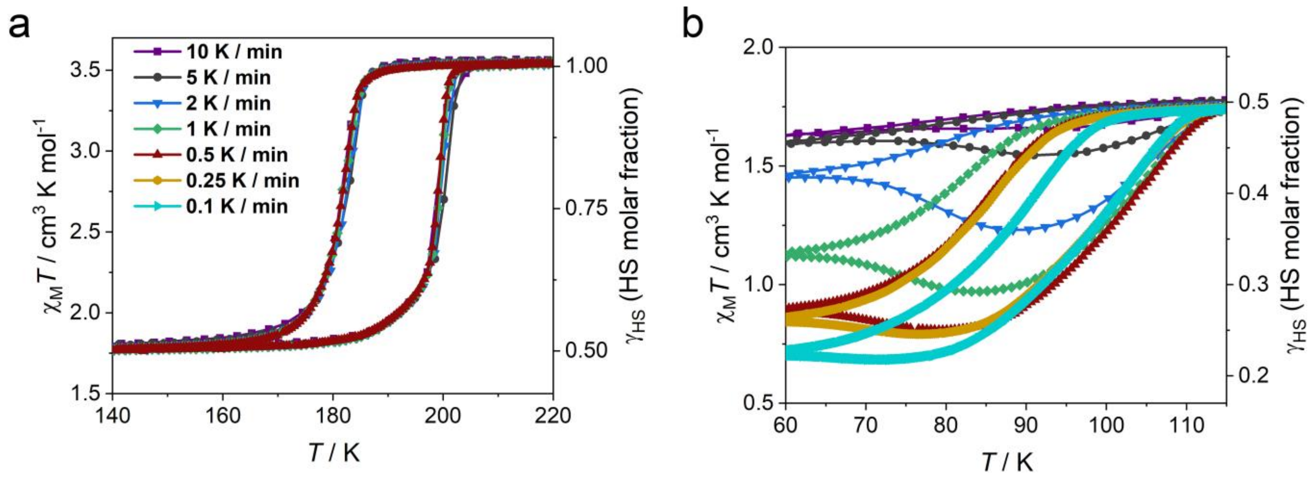

Figure S26. The enlarged view of the $\chi_M T$ of the guest-saturated sample with maintaining 8:2 H_2O-CH_3OH ratio product in the interval of 140−220 (**a**) and 60−115 K (**b**).

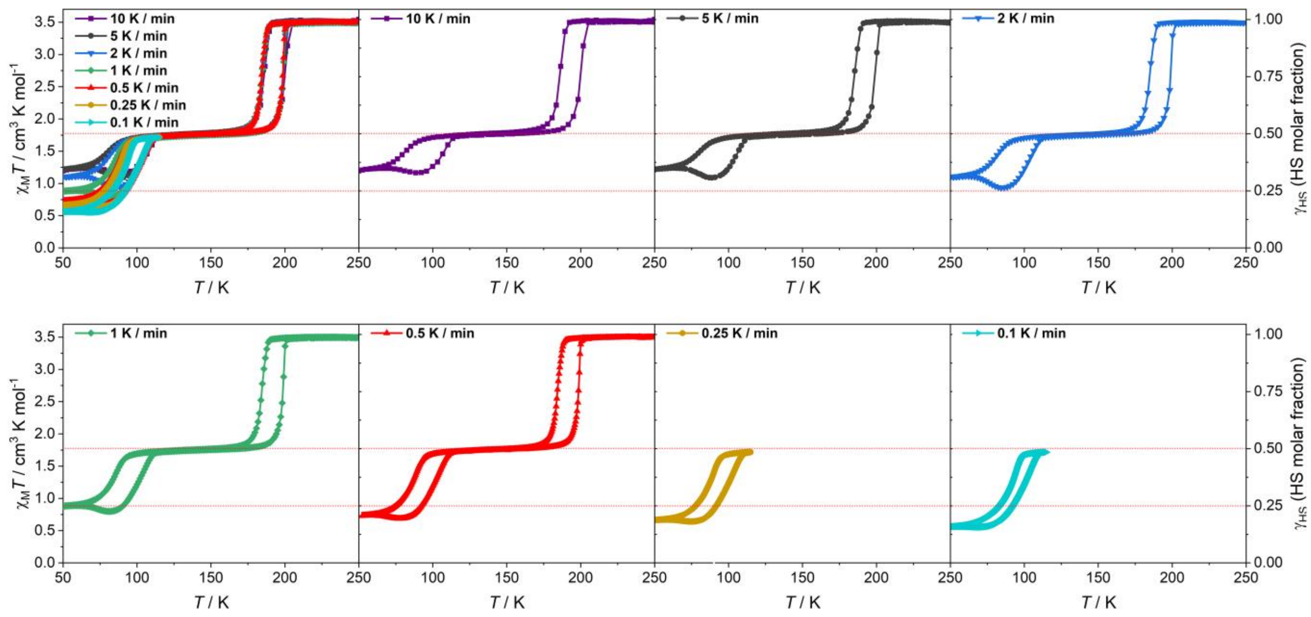

Figure S27. Temperature-dependent $\chi_M T$ values of the guest-saturated sample with 8:2 H_2O-CH_3OH ratio after in-situ heating at 300 K for 10 min.

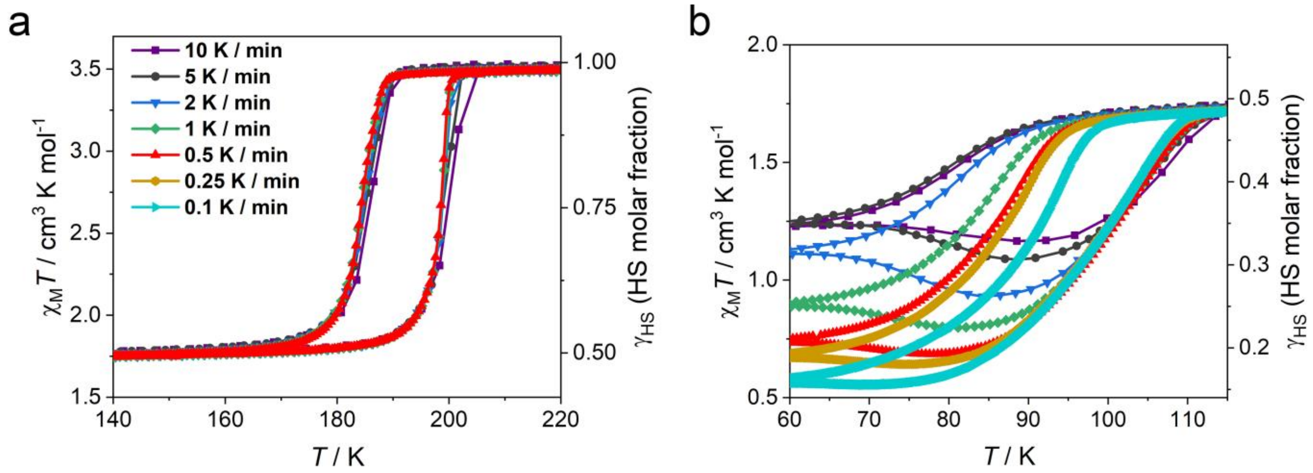

Figure S28. The enlarged view of the $\chi_M T$ of the guest-saturated sample with partial guest losing 8:2 H_2O-CH_3OH ratio product in the interval of 140−220 (**a**) and 60−115 K (**b**).

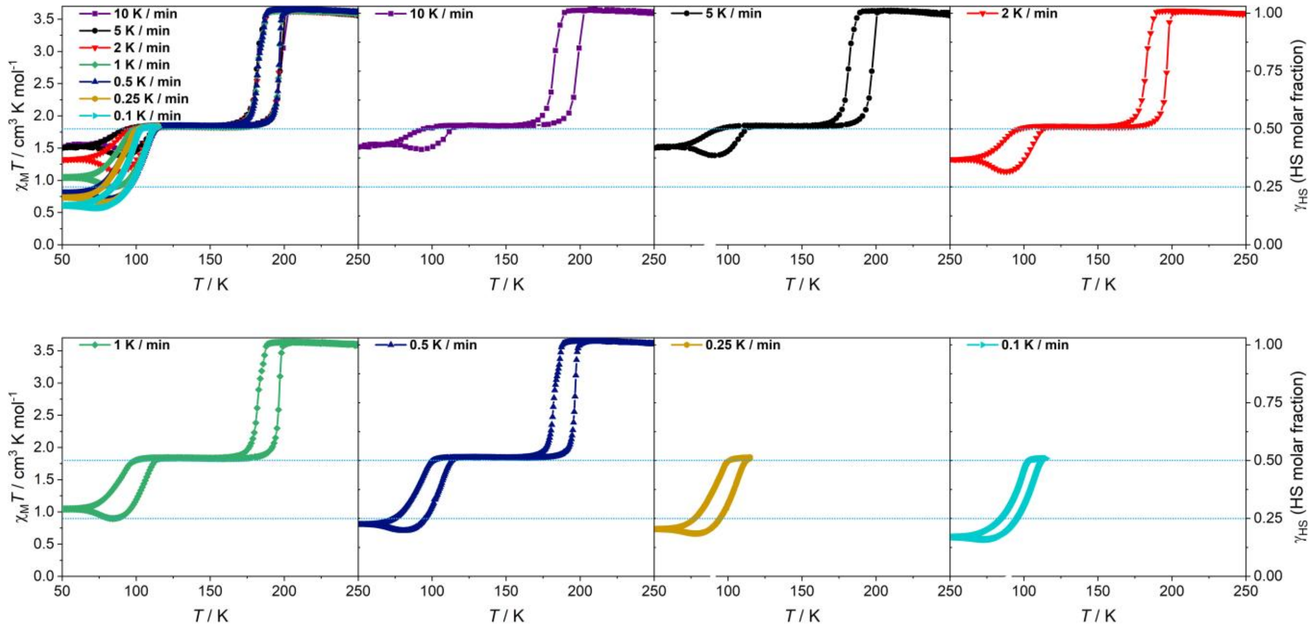

Figure S29. Temperature-dependent $\chi_M T$ values of the guest-saturated sample with 10:0 H_2O-CH_3OH ratio at an ambient temperature of 250 K.

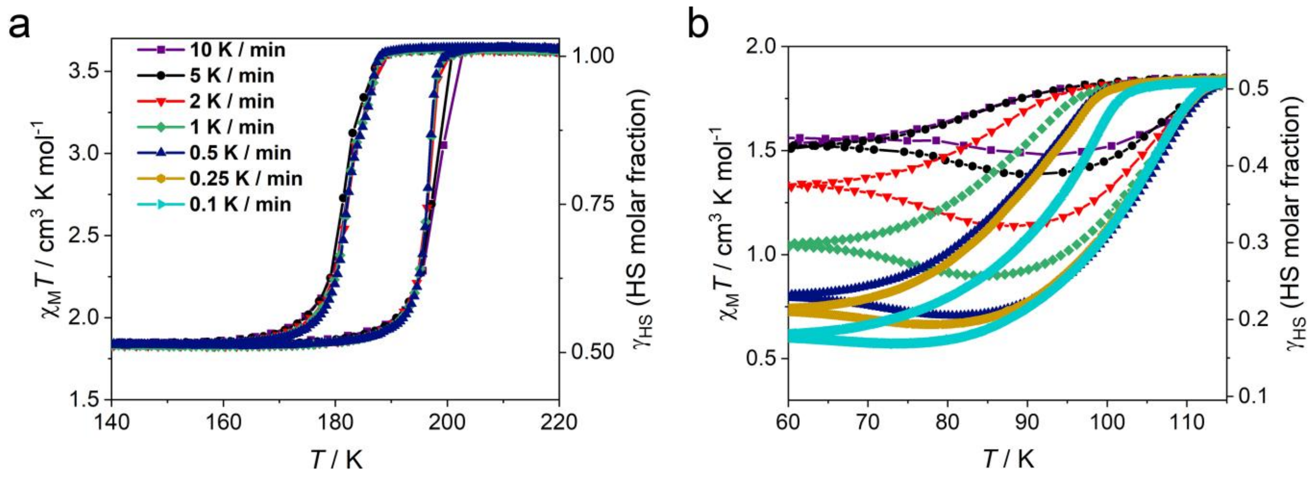

Figure S30. The enlarged view of the $\chi_M T$ of the guest-saturated sample with maintaining 10:0 H_2O-CH_3OH ratio product in the interval of 140−220 (**a**) and 60−115 K (**b**).

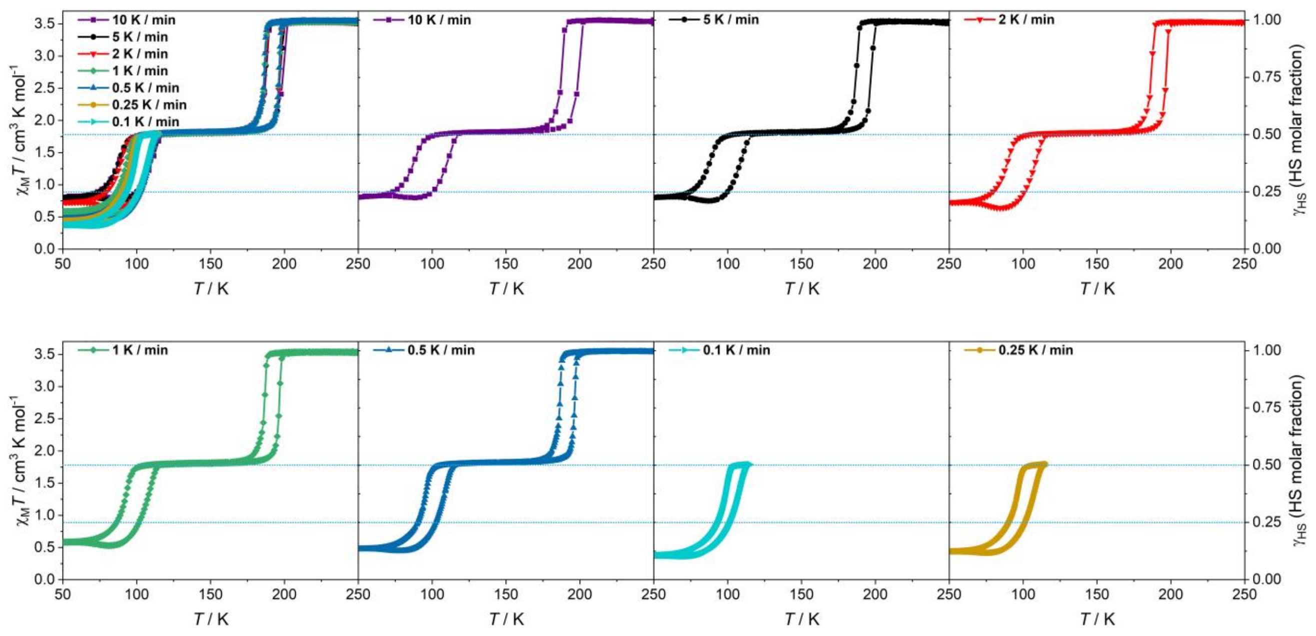

Figure S31. Temperature-dependent $\chi_M T$ values of the guest-saturated sample with 10:0 H_2O-CH_3OH ratio after in-situ heating at 300 K for 10 min.

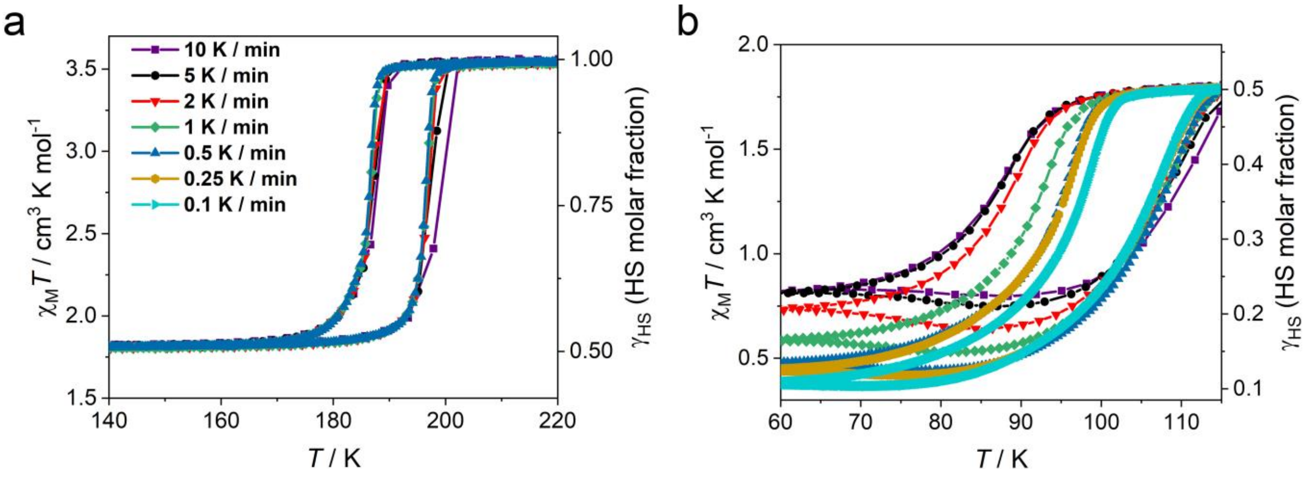

Figure S32. The enlarged view of the $\chi_M T$ of the guest-saturated sample with partial guest losing 10:0 H_2O-CH_3OH ratio product in the interval of 140−220 (**a**) and 60−115 K (**b**).

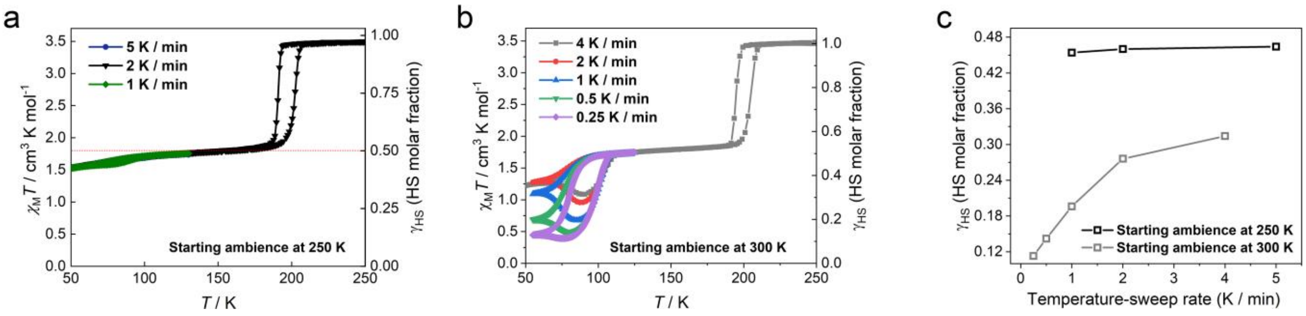

Figure S33. Temperature-dependent $\chi_M T$ values of the guest-saturated samples with 2:8 H_2O-CH_3OH ratio. The fresh sample was measured at an ambient temperature of 250 K to start the cooling-warming cycles (1, 2, 5 K min^{-1}, **a**). Then the sample was warmed up to 300 K and held for 10 min, followed by cooling down to 250 K to measure in the cooling-warming cycles (0.25, 0.5, 1, 2, 4 K min^{-1}, **b**). **c**, The temperature-sweep rate vs HS Fe^{II} molar fraction (γ_{HS}) for the sample with 2:8 H_2O-CH_3OH ratio starting different ambiences.

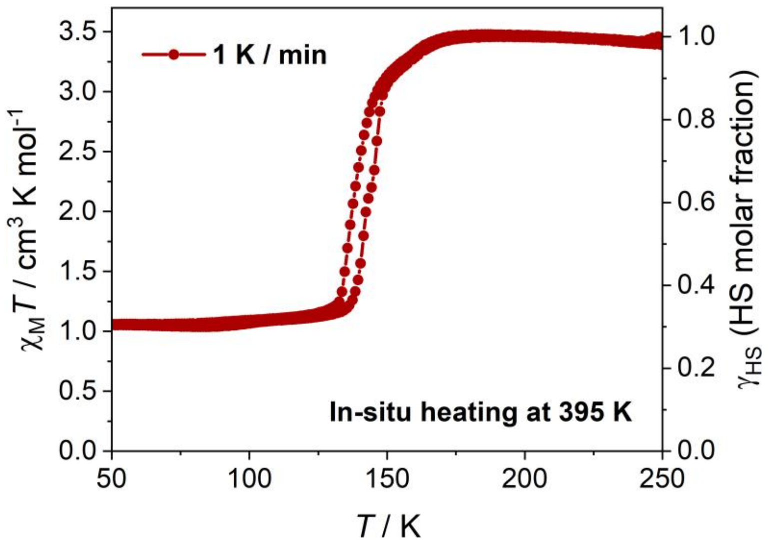

Figure S34. Temperature-dependent $\chi_M T$ and γ_{HS} values of the guest-saturated samples with the 2:8 H_2O-CH_3OH ratio under in situ heating at 395 K for 1 hour (the fourth cooling-warming cycle).

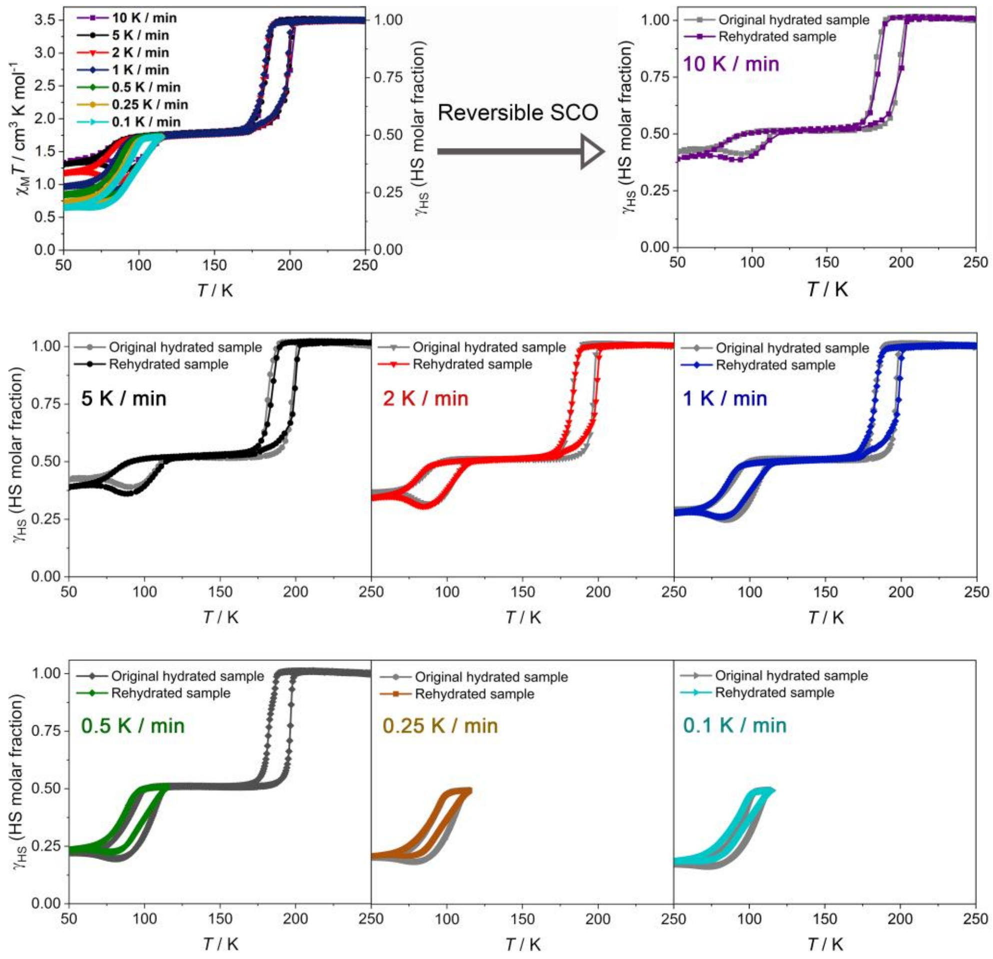

Figure S35. Temperature-dependent γ_{HS} values of the original hydrated and sample and rehydrated sample after heating at 395 K under various scan rates.

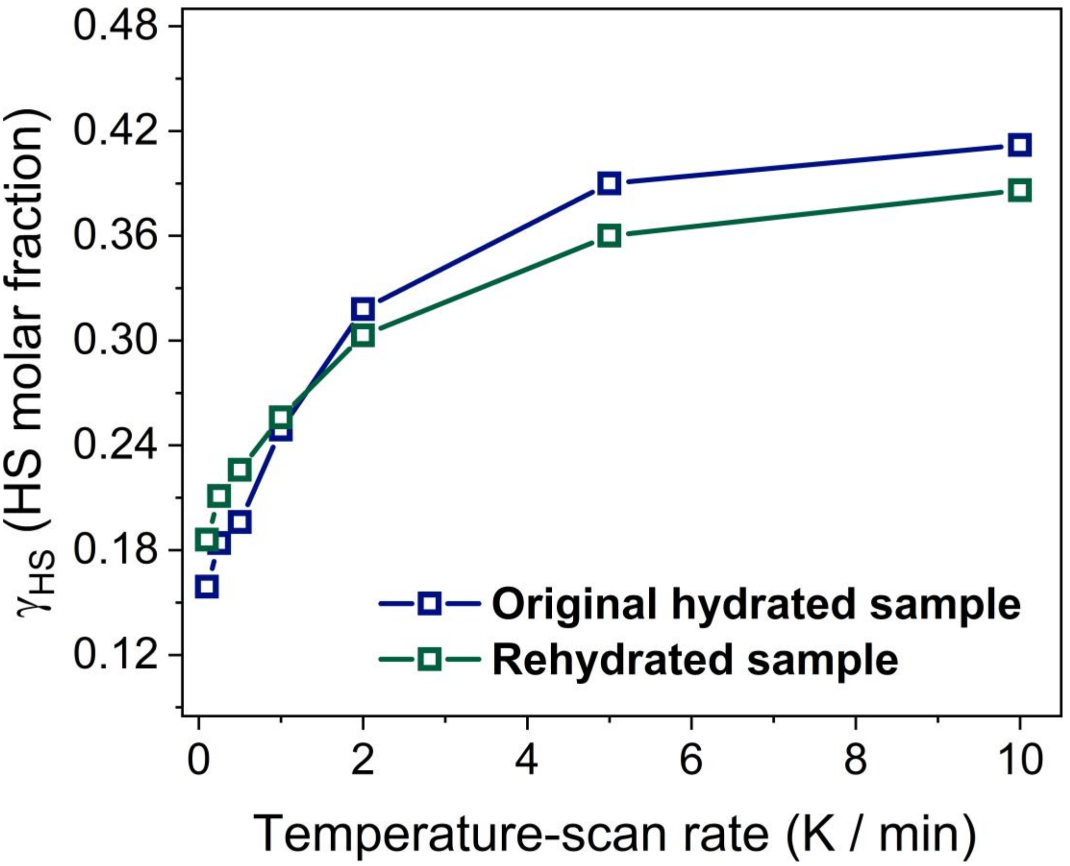

Figure S36. The temperature-sweep rate vs residual HS Fe^{II} molar fraction (γ_{HS}) for the original hydrated sample and the rehydrated sample after heating at 395 K starting at an ambient temperature of 250 K.

Table S1. Crystal data and structural refinements for 1·3H_2O·3/2CH_3OH.

	1·3H_2O·3/2CH_3OH					
Temperature/K	293	190	150	130	120	85
Formula			$C_{27.5}H_{30.5}FeN_{12}O_{4.5}Pd$			
*M*r/g mol^{-1}			763.39			
Crystal size/mm			0.05×0.08×0.03			
Wavelength/Å			0.77484			
Space group	$P\bar{1}$	$P\bar{1}$	$P\bar{1}$	$P\bar{1}$	$P\bar{1}$	$P\bar{1}$
Crystal color	yellow	red	red	red	red	red
Crystal system	triclinic	triclinic	triclinic	triclinic	triclinic	triclinic
a/Å	7.4570(15)	7.2880(15)	7.2770(15)	7.2810(15)	7.2780(15)	7.2600(15)
b/Å	14.711(3)	14.504(3)	14.509(3)	14.499(3)	14.500(3)	14.476(3)
c/Å	15.442(3)	15.422(3)	15.381(3)	15.355(3)	15.347(3)	15.344(3)
α/°	104.19(3)	104.70(3)	104.67(3)	104.60(3)	104.59(3)	104.57(3)
β/°	99.92(3)	100.07(3)	99.99(3)	99.89(3)	99.88(3)	99.87(3)
γ/°	90.28(3)	90.33(3)	90.32(3)	90.32(3)	90.32(3)	90.32(3)
Volume/Å^3	1613.5(6)	1550.4(6)	1545.1(6)	1543.3(6)	1542.0(6)	1535.6(6)
Z	2	2	2	2	2	2
D_c / g cm^{-3}	1.571	1.635	1.641	1.643	1.644	1.651
μ / mm^{-1}	1.062	1.105	1.109	1.110	1.111	1.116
F(000)	775.0	775.0	775.0	775.0	775.0	775.0
θ range/°	2.864 to 50.052	2.776 to 50.054	2.782 to 50.044	2.906 to 50.044	2.906 to 50.054	2.91 to 50.054
Data/restraints/parameters	5202/422/424	5053/721/424	4992/25/424	4930/403/424	4910/25/424	3590/375/400
Goodness-of-fit on F^2	1.070	1.034	1.038	1.029	1.033	1.370
Reflections collected	10053	9820	9540	9249	9169	5359
R_1 [I ≥ 2σ(I)]	0.0675	0.0692	0.0699	0.0745	0.0761	0.1307
wR_2 [all data]	0.1997	0.1962	0.2012	0.2148	0.2183	0.3547
Largest diff. peak and hole/e.Å^{-3}	1.89/-1.41	1.99/-1.71	1.96/-1.66	2.34/-2.05	2.20/-1.98	4.47/-1.92

Table S2. Selected bond lengths and angles for 1·3H_2O·3/2CH_3OH at different temperatures.

	293 K	190 K	150 K	130 K	120 K	85 K
Fe1−N4	2.161(6)	1.981(5)	1.967(5)	1.965(6)	1.972(6)	1.986(12)
Fe1−N5	2.156(5)	1.947(5)	1.952(6)	1.946(6)	1.946(6)	1.966(14)
Fe1−N6	2.152(5)	1.950(5)	1.950(5)	1.942(6)	1.949(6)	1.943(14)
Fe2−N7	2.148(5)	2.146(5)	2.149(6)	2.151(6)	2.152(6)	2.130(14)
Fe2−N8	2.177(5)	2.161(5)	2.159(6)	2.157(6)	2.168(6)	2.121(14)
Fe2−N12	2.187(5)	2.179(5)	2.175(5)	2.177(5)	2.178(6)	2.164(12)
$N5^{1}$−Fe1−$N4^{1}$	90.4(2)	90.3(2)	89.9(2)	90.2(2)	90.2(2)	91.4(5)
$N5^{1}$−Fe1−N4	89.6(2)	89.7(2)	90.1(2)	89.8(2)	89.8(2)	88.6(5)
N5−Fe1−$N4^{1}$	89.6(2)	89.7(2)	90.1(2)	89.8(2)	89.8(2)	88.6(5)
N5−Fe1−N4	90.4(2)	90.3(2)	89.9(2)	90.2(2)	90.2(2)	91.4(5)
$N6^{2}$−Fe1−N4	88.0(2)	91.2(2)	88.9(2)	91.3(2)	88.9(2)	91.4(5)
$N6^{3}$−Fe1−N4	92.0(2)	88.8(2)	91.1(2)	88.7(2)	91.1(2)	88.6(5)
$N6^{2}$−Fe1−$N4^{1}$	92.0(2)	88.8(2)	91.1(2)	88.7(2)	91.1(2)	88.6(5)
$N6^{3}$−Fe1−$N4^{1}$	88.0(2)	91.2(2)	88.9(2)	91.3(2)	88.9(2)	91.4(5)
$N6^{2}$−Fe1−N5	89.6(2)	89.8(2)	89.9(2)	90.1(2)	90.0(2)	89.7(5)
$N6^{3}$−Fe1−N5	90.4(2)	90.2(2)	90.1(2)	89.9(2)	90.0(2)	90.3(5)
$N6^{3}$−Fe1−$N5^{1}$	89.6(2)	89.8(2)	89.9(2)	90.1(2)	90.0(2)	89.7(5)
$N6^{2}$−Fe1−$N5^{1}$	90.4(2)	90.2(2)	90.1(2)	89.9(2)	90.0(2)	90.3(5)

Symmetry codes: ^{1} $-x, 1-y, -z$; ^{2} $-1+x, +y, +z$; ^{3} $1-x, 1-y, -z$.

Table S3. Selected structural parameters for 1·3H_2O·3/2CH_3OH at different temperatures.

Compound	**1·3H_2O·3/2CH_3OH**					
Parameter	293 K	190 K	150 K	130 K	120 K	85 K
<Fe1−N>[a]/Å	2.156(5)	1.959(5)	1.956(5)	1.951(6)	1.956(6)	1.965(13)
<Fe2−N>[a]/Å	2.170(5)	2.162(5)	2.161(5)	2.162(6)	2.166(6)	2.138(13)
∑Fe1[b]	11.2(2)	6.8(2)	5.2(2)	6.4(2)	5.2(2)	12.4(5)
∑Fe2[b]	5.6(3)	11.72(8)	12.0(2)	13.6(2)	13.2(2)	14.4(5)
d_{N-O}[c]	2.904(4)	2.817(3)	2.822(3)	2.824(4)	2.821(3)	2.850(3)

[a] The average Fe–N bond lengths (Å);

[b] Octahedral distortion parameters (°);

[c] The distance of hydrogen bonds between uncoordinated nitrogen atom of 1,2,4-triazole in the prentrz ligand and oxygen atom of adjacent H_2O molecule.

References

1. Scott, H. S., Ross, T. M., Moubaraki, B., Murray, K. S. & Neville, S. M. (2013). Spin crossover in polymeric materials using schiff base functionalized triazole ligands. Eur. J. Inorg. Chem. 803-812.
2. Xue, J-P., Hu, Y., Zhao, B., Liu, Z-K., Xie, J., Yao, Z-S. & Tao J. (2022). A spin-crossover framework endowed with pore-adjustable behavior by slow structural dynamics. Nat. Commun., 13, 3510.
3. Minor, W., Cymborowski, M., Otwinowski, Z. & Chruszcz, M. (2006). HKL-3000: the Integration of Data Reduction and Structure Solution – from Diffraction Images to an Initial Model in minutes. *Acta. Cryst. D*. **62**, 859–866.
4. Sheldrick, G. M. (2015). Crystal structure refinement with SHELXL. *Acta Cryst. C*. **71**, 3–8.
